# The outcome of conversion total hip arthroplasty following acetabular fractures: a systematic review and meta-analysis of comparative studies

**DOI:** 10.1186/s13018-024-04561-x

**Published:** 2024-01-20

**Authors:** Farhad Shaker, Sina Esmaeili, Mobina Taghva Nakhjiri, Alireza Azarboo, Seyyed Hossein Shafiei

**Affiliations:** Orthopaedic Department, Orthopaedic Subspecialty Research Center (OSRC), Sina University Hospital, Tehran University of Medical Sciences, Tehran, Iran

**Keywords:** Acetabular fracture, Conversion THA, Total hip arthroplasty, Secondary THA, Treatment failure

## Abstract

**Background:**

Conversion total hip arthroplasty (THA) is considered the main treatment plan for patients with first-line treatment failure of acetabulum fracture. This meta-analysis aims to assess the effect of the type of initial treatment and timing of surgery on the outcomes of conversion THA.

**Methods:**

Using PRISMA guidelines, MEDLINE/PubMed, Scopus, Web of Science, and CENTRAL Cochrane were searched for articles published before October 14, 2022. Comparative studies investigating the outcome of THA following treatment failure of acetabular fracture were included. These articles were categorized into three groups, and the outcomes of treatment plans in each group were compared: (A) primary THA vs. conversion THA, (B) THA following conservative treatment vs. THA following ORIF, and (C) acute THA vs. delayed THA following prior treatment failure. Review Manager (RevMan, version 5.3) software was utilized to perform the statistical analysis.

**Results:**

Twenty-four comparative studies met the inclusion criteria (reported the data of 13,373 patients). Concerning group (A), the following complications were significantly higher in conversion THA: Infection (OR [95% CI] 3.19 [2.12, 4.79]; *p* value < 0.00001), dislocation (OR [95% CI] 4.58 [1.56, 13.45]; *p* value = 0.006), heterotopic ossification (OR [95% CI] 5.68 [3.46, 9.32]; *p* value < 0.00001), and Revision (OR [95% CI] 2.57 [1.65, 4.01]; *p* value < 0.00001). Postoperative HHS (SMD [95% CI] − 0.66 [− 1.24, − 0.08]; *p* value = 0.03) was significantly lower and operation time (SMD [95% CI] 0.88 [0.61, 1.15]; *p* value < 0.00001), blood loss (SMD [95% CI] 0.83 [0.56, 1.11]; *p* value < 0.00001), and bone graft need (OR [95% CI] 27.84 [11.80, 65.65]; *p* value < 0.00001) were significantly higher in conversion THA. Regarding group (B), bone graft need (OR [95% CI] 0.48 [0.27, 0.86]; *p* value = 0.01) was considerably higher in patients with prior acetabular fracture conservative treatment, while other outcomes were comparable. Respecting group (C), there were no significant differences in analyzed outcomes. However, systematically reviewing existing literature suggested a higher incidence rate of DVT following acute THA.

**Conclusion:**

There were significantly higher postoperative complications and lower functional outcomes in conversion THA compared to primary THA. While complications and functional outcomes were comparable between ORIF and the conservative groups, the bone graft need was significantly higher in the conservative group. There were no significant differences between aTHA and dTHA. These results can assist surgeons in designing treatment plans based on each patient’s clinical situation.

*Prospero registration code*: CRD42022385508.

*Level of evidence*: III/IV.

**Supplementary Information:**

The online version contains supplementary material available at 10.1186/s13018-024-04561-x.

## Introduction

Acetabular fracture is considered to be a somewhat common high-energy trauma, accounting for an incidence of nearly 4 million persons/year worldwide [1]. Multiple treatment options have been suggested to treat acute acetabular fractures, including conservative approach, open reduction and internal fixation (ORIF), and total hip arthroplasty (THA). However, no generally approved option has been recommended so far [2]. The major goals of fixation are blood supply preservation, stability, and anatomical restoration. Despite these, osteoarthritis and femoral head osteonecrosis are among the most prevalent late complications of acetabular fracture treatment. Approximately 13 to 44% of patients undergoing acetabular fracture surgery eventually suffer from subsequent hip problems requiring additional therapies. About 8.5% of these complications develop within two years of first-line management [3, 4].

Till now, conversion THA (cTHA) remains the gold standard procedure to manage post-fixation complications leading to failure due to significant pain relief and quality of life improvement. However, orthopedic surgeons have to fight off some technical challenges while performing cTHA rather than primary THA (pTHA) (like bone graft need, acetabular reconstruction, and Dealing with the previous implants) [5–7]. Therefore, it is assumed to have a higher complication rate and worse functional outcomes. Comparing the outcome of cTHA with pTHA is still a hot topic among trauma researchers [8–10].

The existing evidence indicated that cTHA outcome might depend on two major conditions: type of first-line management (ORIF or conservative) and time between initial fixation and subsequent THA (acute or delayed). Some previous investigations concluded that there is no difference between groups that have undergone early surgical management and those that have undergone conservative management regarding hip survival rate [11], while others did not think the same [12]. It is also a fundamental matter of debate comparing acute and delayed THA following acetabular fracture [13].

Although some original studies made an endeavor to evaluate the outcome of conversion THA, there is yet a high demand to perform a comprehensive review pooling all relevant findings together to build a consensus on whether cTHA is efficient. This systematic review aimed to answer the following research questions: (1) Does the type of THA (cTHA vs. pTHA) impact the rates of complications, functional status, and intraoperative outcomes? (2) Does the type of first-line management for acetabular fracture (ORIF vs. conservative management) affect the outcomes of subsequent cTHA? (3) Does the timing of THA (acute vs. delayed) following acetabular fracture influence its outcomes? These questions will be addressed through a comprehensive review of randomized control trials, cohort studies, and case series examining these specific comparisons and outcomes.

## Material and method

The PRISMA (Preferred Reporting Items for Systematic Reviews and Meta-Analyses) guidelines served as a framework for our study’s phases [14]. The review protocol has also been registered in Prospero (registration code: CRD42022385508).

### Searching phase

We searched for studies on THA after failed treatment of acetabulum in the electronic databases MEDLINE/PubMed, Scopus, Web of Science (WOS), and CENTRAL Cochrane, published until October 14, 2022. A search strategy was written for each database using the keywords “Acetabulum,” “Acetabular fracture,” “Fracture fixation,” “Open reduction and internal fixation,” “ORIF,” “Total hip arthroplasty,” “Conversion total hip arthroplasty,” and “THA.” We also searched the reference section and the “cited by” papers of the qualifying articles for adding probable relevant studies. Our complete search strategy is presented in the (Additional file 1: Table S1).

### Eligibility criteria and paper selection

We included comparative studies involving patients who underwent THA due to failed treatment of the acetabular fracture with a minimum mean or median follow-up of two years. Acceptable study designs were prospective and retrospective cohort, case–control, randomized controlled trials (RCTs), and non-randomized clinical trials. We did not limit the publication year for the included studies. Review articles, case reports, congress abstracts, letters, non-English publications, non-human models, and articles without a group of patients with failed treatment of acetabular fracture were excluded. The criteria for including studies based on PICOS (participants, interventions, comparison, outcomes, and study type) are outlined in Table 1.

**Table 1:** PICOS criteria for eligibility

P (Participants)	Patients who underwent total hip arthroplasty due to failed treatment of the acetabular fracture
I (Intervention)	Total hip arthroplasty
C (Comparison)	Group A: comparison outcome of total hip arthroplasty following failed treatment of acetabular fracture with patients who underwent primary total hip arthroplasty Group B: comparison outcome of total hip arthroplasty following failed first-line open reduction and internal fixation management with conservative management Group C: comparison outcome of delayed total hip arthroplasty following failed treatment of acetabular fracture with patients who underwent acute total hip arthroplasty
O (Outcomes)	Complications, revision, functional outcomes, operation time, blood loss, and bone graft need
S (Study type)	Cohort study, prospective study, retrospective study, case–control, randomized controlled trials, and non-randomized clinical trials

We imported all of the studies into the Covidence online tool [15]. After removing duplicates, two researchers (FS, MT) independently screened the remaining articles based on the title and abstract to determine if they met the inclusion criteria. Subsequently, during the full-text screening phase, two previous reviewers, FS and MT, individually re-assessed each of the selected articles. In the event of a disagreement, a third reviewer (SHS) intervened and resolved it.

### Data extraction

The pilot extraction was conducted during a consensus meeting with the corresponding authors and inconsistencies were addressed. Three researchers (FS, MT, SE) independently extracted and collected the data of the included studies in a spreadsheet. We obtained the following data from included articles: publication year, first author’s name, country, study design, study and control population, age, gender, comorbidity, injury type, type of acetabular fracture, first treatment approach, THA approach, diagnosis for THA, Interval between two operations, follow-up duration, lost to follow-up, operation time, blood loss, transfusion need, bone graft need, leg length discrepancy (LLD), quality of life and functional outcome measures, length of hospital stay (LOS), readmission, reoperation, mortality, loosening, heterotopic ossification (HO), dislocation, postop fracture, deep vein thrombosis (DVT), infection and other complications.

### Quality assessment

Two separate reviewers evaluated each study’s quality. The Newcastle–Ottawa Scale (NOS) was employed to evaluate the quality of observational studies [16]. The NOS evaluates a study based on three primary characteristics: group selection, comparability, and outcome evaluation. Studies having a score greater than six were classified as high quality. Scores of 5 or 6 were considered to be of moderate quality. Articles scored less than five were defined as a low-quality study.

### Statistical analysis

Alongside our previously mentioned goals, eligible articles were classified into three groups based on the comparison group. In Group A, we analyze the outcome of THA after failed treatment of acetabular fracture (cTHA) with patients who underwent primary THA (pTHA) due to various diagnoses such as arthritis and necrosis. Group B consists of papers that compare the outcomes of patients who underwent cTHA following first-line ORIF or conservative management. Group C comprises the articles comparing acute THA vs. delayed cTHA following an acetabulum fracture treatment.

Review Manager 5.3 software (Cochrane Collaboration, Software Update, Oxford, United Kingdom) was used for the meta-analysis. We conducted a meta-analysis if three or more papers reported a specific outcome. Regarding the dichotomized outcomes such as complications, the Mantel–Haenszel model was utilized, and the odds ratio (OR) and its 95% CI were reported. Inverse variance model was applied for the continuous outcomes, like the postoperative Harris Hip Score, and Standardized Mean Difference (SMD) [95% CI] was reported. Random-effect models were employed when the *I*^2^ was greater than 50% (Heterogenous data). A *p* value less than 0.05 is considered statistically significant. Leave-one-out meta-analysis is employed technique for conducting sensitivity analysis, particularly for non-significant outcomes. Egger's Regression test and Begg's funnel plot were utilized to evaluate publication bias on the outcomes with the highest number of studies through the utilization of comprehensive meta-analysis (CMA) software.

## Result

### Study selection

**Fig. 1:**
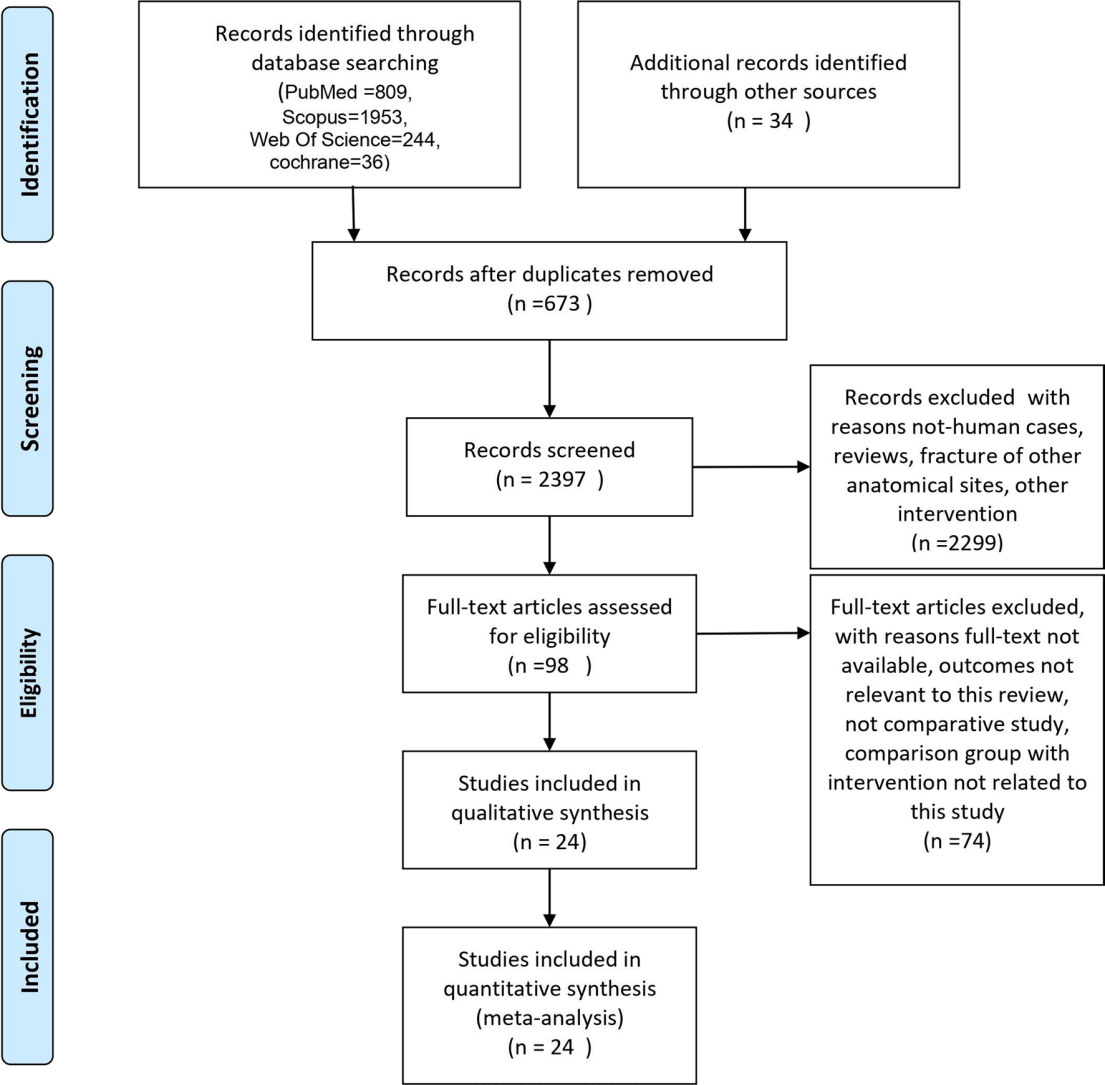
PRISMA flowchart

A total of 3036 citations were found in our initial search. After omitting duplicates, 2397 articles remained to be screened. Title/Abstract screening was done, and 98 articles were considered relevant. Following checking full-texts, 75 studies were removed. Eventually, 24 [9, 11, 13, 17–37] investigations (including 13,373 patients) were considered eligible to enter this systematic review (Fig. 1).

### Quality assessment

Four of the included articles were conducted prospectively. NOS was utilized to assess the methodological quality of the studies. None of them had a high risk of bias (NOS score < 7) (Additional file 1: Fig. S1).

### Study characteristics

Our meta-analysis included a total of 7713 men and 5432 women, with the mean population age ranging from 17 to 104. The most common types of acetabular fracture included posterior wall (PW) [9, 11, 19, 22, 25, 28–30, 34, 36], PW + Posterior Column (PC) [25, 33], Anterior Column (AC) + posterior hemi-transverse [13, 31], both columns [13, 18, 22, 33], and PW + T-type [21, 35]. Studies were categorized into 3 groups, with distinct meta-analyses performed on each: Conversion vs. primary THA (549 patients vs. 12,138 patients) [9, 17, 24, 25, 27–29, 33, 34], ORIF vs. conservative first-line treatments (290 patients vs. 175 patients) [11, 17, 20, 21, 23, 29–32, 36, 37], and acute vs. delayed THA (160 patients vs. 165 patients) [13, 18, 19, 22, 26, 27, 35] (Table 2).

**Table 2:** Baseline characteristics of the eligible studies

Author, year	Country	Groups	Sample size	Age, years	Male:Female	Fracture type, n	Follow-up duration
Aali Rezaei et al. [9]	USA	cTHA/pTHA	cTHA: 72 pTHA: 215	cTHA: 57 (25–89) pTHA: 58 (25–87)	cTHA: 37:35 pTHA: 109:106	PW: 21, PC: 3, AC: 3, Transverse: 2, PC/PW: 12, Transverse/PW: 11, AC/posterior hemi-transverse: 9, Both columns: 8, T- type: 1	cTHA: 34.8 (12–150) months pTHA: 36.72 (12–155) months
Bellabarba et al. [17]	USA	cTHA/pTHA ORIF/Conservative	cTHA: 30 (ORIF: 15 Conservative: 15) pTHA: 184	cTHA: 51 (26–86) pTHA: 52 (20–84) ORIF: 50 (26–86) Conservative: 53 (29–85)	cTHA: 14:16 pTHA: 82:102 ORIF: 10:5 Conservative: 4:11	NA	cTHA: 63 (24–140) months pTHA: 104 (78–126) months
Lizaur-utrilla et al. [25]	Spain	cTHA/pTHA	cTHA: 24 pTHA: 48	cTHA: 56.4 ± 13.8 pTHA: 57.4 ± 12.6	cTHA: 19:5 pTHA: 38:10	PW 8, AC 2, PW and column 9, T fracture 5	cTHA: 100.8 (60–180) months pTHA: 90 (60–144) months
Schnaser et al. [33]	USA	cTHA/pTHA	cTHA: 17 pTHA: 21	cTHA: 69 (60–81) pTHA: 70	cTHA: 13:4 pTHA: NM	Associated both column 5, AC and posterior hemi-transverse 2, AC 1, PW 2, PC and PW 5, T-type 2	77 ± 33 months
Scott et al. [34]	UK	cTHA/pTHA	cTHA: 49 pTHA: 98	cTHA: 57 (25–87) pTHA: 55.6 (17–81)	cTHA: 33:16 pTHA: 66:32	PW 17, PC 0, AW 1, AC 1, Transverse 4, T-shaped 5, PC and wall 4, Transverse PW 8, AC, posterior hemi-transverse 4, Both columns 3, Unknown 2	cTHA: 9.1y (0.5–23) pTHA: 7.6y (4–10)
McGowan et al. [28]	USA	cTHA/pTHA	cTHA: 30 pTHA: 20	cTHA: 51.7 ± 11.4 pTHA: 55.3 ± 12.7	cTHA: 15:15 pTHA: 10:10	PW: 8, AW:1, AC:1, Transverse: 2, AC posterior hemi-transverse: 2, PC PW: 2, Transverse PW: 4, T-type: 4, Both columns: 6	cTHA: 36.4 (2–51) months pTHA: 7.4 months
Manirajan et al. [27]	USA	cTHA/pTHA	cTHA: 196 pTHA: 11,421	cTHA: NM pTHA: 67.3	cTHA: NM pTHA: 6678:4743	NA	2-year
Morison et al. [29]	Canada	cTHA/pTHA ORIF/Conservative	cTHA: 74 (ORIF: 58 Conservative: 16) pTHA: 74	cTHA: 51 (25–75) pTHA: 52 (30–81)	cTHA: 50:24 pTHA: 50:24	cTHA: AC 2, PC 5, PW 23, transverse 5, anterior + posterior hemi-transverse 4, both columns 12, PC + PW 8, transverse + PW 7, T-type 8	cTHA: 8 (2–23) years pTHA: 10 (2–24) years
Lee et al. [24]	Korea	cTHA/pTHA	cTHA: 57 pTHA: 57	cTHA: 52.5 ± 13.6 pTHA: 52.1 ± 14.6	cTHA: 31:26 pTHA: 30:27	NA	cTHA:7.8 ± 2.4 years pTHA:7.8 ± 2.8 years
Garcia-Rey et al. [21]	Spain	ORIF/conservative	ORIF: 29 Conservative: 49	ORIF: 52.9 (23–78) Conservative: 59.3 (27–84)	ORIF: 22:7 Conservative: 26:23	ORIF: No radiograph: 1, AW: 0, AC: 1, PW: 2, PC: 3, Transverse: 0, Both columns: 4, PC/ PW: 6, Transverse/PW–T-type:9, T-type: 1 Conservative: No radiograph: 8, AW: 2, AC: 0, PW: 5, PC: 6, Transverse: 8, Both columns: 6, PC/ PW: 6, Transverse/ PW–T-type: 4, T-type: 2	ORIF: 10.2 years (5 to 20) Conservative: 11.7 years (5 to 23)
Gavaskar et al. [11]	India	ORIF/conservative	ORIF: 24 Conservative: 20	ORIF: 47 ± 9 Conservative: 49 ± 9	ORIF: 18:6 Conservative: 13:7	ORIF: PW: 8, PC: 2, AW: 0, AC: 1, Transverse: 5, PW + PC: 2, Transverse + PW: 3, AC + posterior hemi-transverse: 1, T type: 3, Both column: 2 Conservative: PW: 1, PC: 3, AW: 1, AC: 3, Transverse: 4, PW + PC: 2, Transverse + PW: 1, AC + posterior hemi-transverse: 1, T-type: 2, Both column: 2	ORIF: 82 ± 117 months Conservative: 85 ± 16 months
Lai et al. [23]	China	ORIF/conservative	ORIF: 19 Conservative: 12	ORIF: 50 ± 10 Conservative: 52 ± 15	ORIF: 13:6 Conservative: 9:3	ORIF: complex: 11, simple:8 Conservative: complex:5, simple:7	6.3 years (range, 3.1–8.4 years)
Ranawat et al. [30]	USA	ORIF/conservative	ORIF: 24 Conservative: 8	ORIF: 49.7 (19–82) Conservative: 47.5 (17–86)	Total: 23:9	ORIF: PW 9, PC 1, transverse 1, both columns 3, + PW 5, PW + transverse 3, T-type 1, AC + posterior hemi-transverse 1 Conservative: PW 4, transverse 2, both columns 1, comminuted AC/AW 1	56.4 (24–116.4) months
Salama et al. [32]	France	ORIF/conservative	ORIF: 17 Conservative: 4	NA	ORIF: 12:5 Conservative: 1:3	Simple fractures 9, Associated fractures 12	26 (24–36) months
Wang et al. [36]	China	ORIF/conservative	ORIF: 21 Conservative: 12	ORIF: 44.9 ± 10.5 Conservative: 45.5 ± 7.2	ORIF: 13:8 Conservative: 8:4	PC 4, PW 7, AC 2, AW 0, transverse 3, PW + PC 3, transverse + PW 6, both columns 4, T-shaped 2, AC + posterior hemi-transverse 3	138 ± 36 (96–204) months
Zhang et al. [37]	China	ORIF/conservative	ORIF: 32 Conservative: 21	ORIF: NM Conservative: 46.6(22–65)	Total: 42:11	ORIF: PW: 14, transverse: 2, both columns: 1, PC + PW: 5, transverse + PW: 10, Conservative: PW: 14, transverse: 2, AC: 1, both columns: 2, PC + PW: 1, transverse + PW: 3	64 (32–123) months
Rommens et al. [31]	Germany	ORIF/conservative	ORIF: 26 Conservative: 3	ORIF: 77 (65–92) Conservative: 81(73–104)	NA	ORIF: AC + posterior hemi-transverse: 12, both columns: 7, AC: 4, PW: 1, T-type: 2	36 (16–73) months
El-bakoury et al. [20]	Egypt	ORIF/conservative	ORIF: 25 Conservative: 15	Total: 46.7 (21–77)	Total: 31:9	NA	50 (16–87) months
Nicol et al. [13]	Canada	Acute/delayed	Acute: 12 Delayed: 14	Acute: 81 ± 7 Delayed: 76 ± 8	Acute: 6:6 Delayed: 8:6	Acute: AC /posterior hemi-transverse: 5, both columns: 3, T-shape: 2, PC /PW: 1, AC: 1, Anterior: 0 Delayed: AC/posterior hemi-transverse: 4, both columns: 5, T shape: 2, PC/PW: 1, AW: 1, Anterior: 1	60 ± 48 months
Garcia et al. [21]	France	Acute/delayed	Acute: 21 Delayed: 39	Acute: 70 (33–95) Delayed: 54 (20–85)	Acute: 17:4 Delayed: 30:9	Acute: PW: 1, PC: 1, transverse: 3, T-type: 4, PW + PC: 3, PW + T: 4, AC + posterior hemi-transverse: 1, both columns: 4 Delayed: T-type: 4, PW + T: 3, AC + posterior hemi-transverse: 5, both columns: 10, PW: 10, AC: 1, PC: 1, transverse: 5	Acute: 42 (24–120) months Delayed: 69.6 (24–132) months
Sermon et al. [35]	Belgium	Acute/delayed	Acute: 64 Delayed: 57	Acute: 78 Delayed: 53	Total: 65:56	Total: PW: 26, transverse: 10, AC: 6, PC: 5, AW: 2, T + PW: 28, both columns: 25, T-shaped: 9, AW + posterior hemi-transverse: 6, PC + PW: 4	30.7 (12–80) months
Carroll et al. [18]	USA	Acute/delayed	Acute: 9 Delayed: 26	Total: 67 (56–89)	Total: 51:42	Both columns: 26, AC + posterior hemi-transverse: 20, PW: 15, transverse/PW: 10, t-type: 7, AC: 6, PC/PW: 5, AW: 2, transverse: 2	60 (24–188) months
Chemaly et al. [19]	Canada	Acute/delayed	Acute: 20 Delayed: 20	Total: 60	NA	Acute: PW: 6, AC: 1, PC: 2, Transverse: 1, T-type: 4, AC + hemi-posterior: 0, Both columns: 3, Transverse + PW: 2, PC + PW: 1, Delayed: PW: 5, AC: 3, PC: 2, Transverse: 1, T-type: 3, AC + hemi-posterior: 1, Associated both columns: 3, Transverse + PW: 1, PC + PW: 1	30 (9–79.2) months
Lont et al. [26]	Finland	Acute/delayed	Acute: 34 Delayed: 9	Acute: 70 (56–87) Delayed: 65 (58–74)	Acute: 24:10 Delayed: 6:3	NA	31.2 (0–108) months

*THA* total hip arthroplasty; *cTHA* conversion THA; *pTHA* primary THA; *PW* posterior wall; *PC* posterior column; *AC* anterior column; *AW* anterior wall; *NA* not available; *n* number

### Group A: conversion vs. primary THA

#### Complications and revisions

**Fig. 2:**
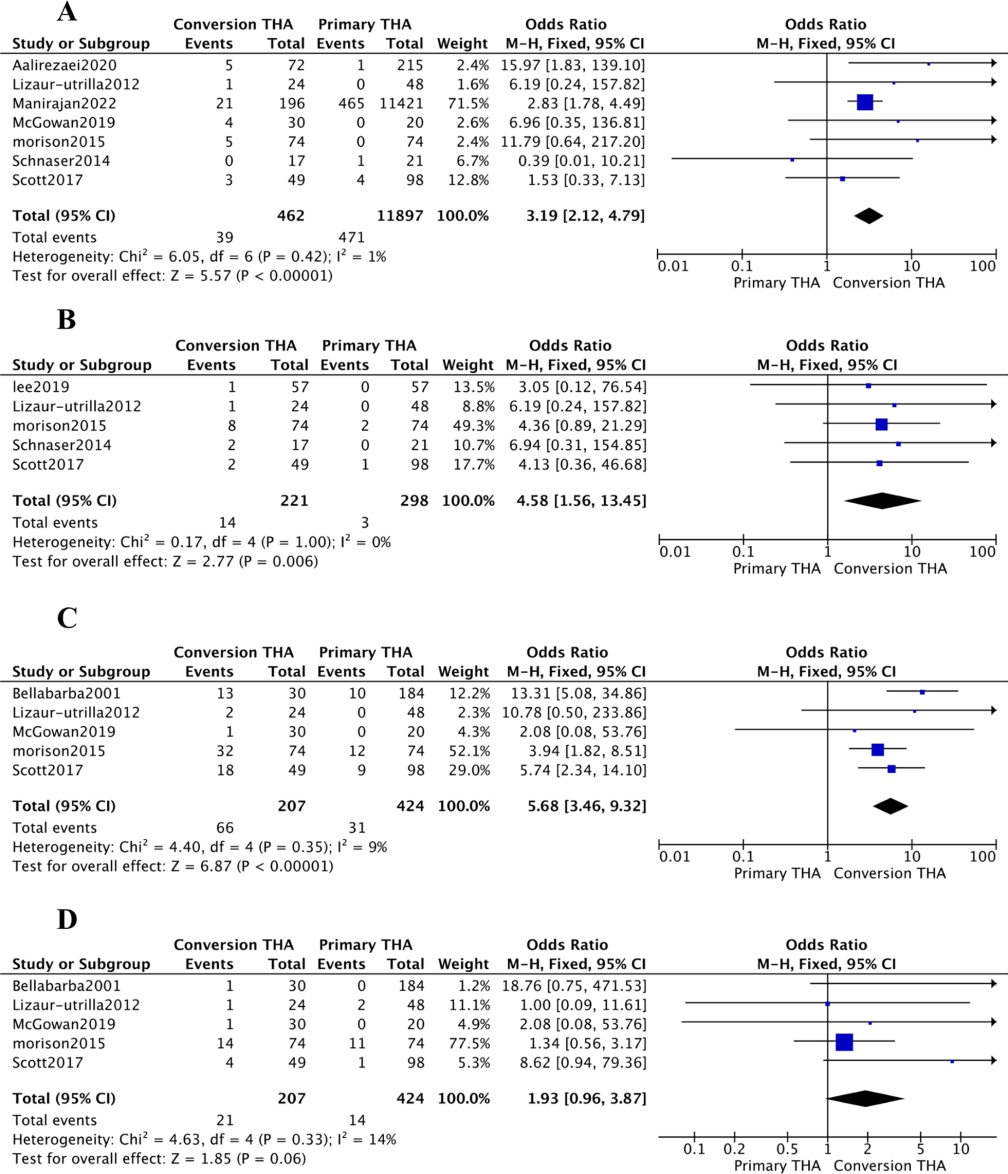
Forest plots demonstrating the rate of infection (**A**), dislocation (**B**), heterotopic ossification (**C**), and implant loosening **D** in those who underwent pTHA versus cTHA

The following postoperative complications were significantly higher within the cTHA group: Infection (OR [95% CI] 3.19 [2.12, 4.79]; *p* value < 0.00001; *I*^2^ = 1%) (Fig. 2A), dislocation (OR [95% CI] 4.58 [1.56, 13.45]); *p* value = 0.006; *I*^2^ = 0%) (Fig. 2B), and heterotopic ossification (OR [95% CI] 5.68 [3.46, 9.32]; *p* value < 0.00001; *I*^2^ = 9%) (Fig. 2C). Implant loosening (OR [95% CI] 1.93 [0.96, 3.87]; *p* value = 0.06; *I*^2^ = 14%) (Fig. 2D) showed no significant correlation with having a failed acetabular fixation. Revision (OR [95% CI] 2.57 [1.65, 4.01]; *p* value < 0.00001; *I*^2^ = 0%) (Additional file 1: Fig. S2) was deemed necessary for a larger proportion of conversion THA patients than primary patients. Aalirezaei et al. [9] reported sciatic nerve damage occurred during performing conversion THA on six patients in the acetabular fracture group (*p* value < 0.001) (Additional file 1: Table S2).

#### Functional outcomes

**Fig. 3:**
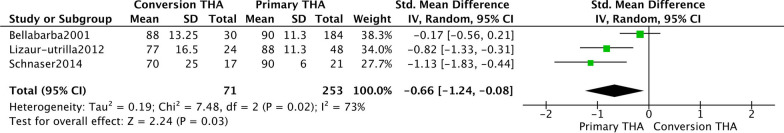
Forest plots demonstrating the port-op HHS in those who underwent cTHA versus pTHA

Postoperative HHS (SMD [95% CI] − 0.66 [− 1.24, − 0.08]; *p* value = 0.03; *I*^2^ = 73%) (Fig. 3) indicates worse patient-reported outcome among patients undergoing conversion THA. Lee et al. [24] estimated the mean UCLA activity for the conversion THA group (4.9 ± 1.9) vs. the primary THA group (5.2 ± 2.0) at the time of the latest follow-up showed better functional outcomes in primary THA (*p* value = 0.404). Scott et al. [34] reported Oxford Hip Score Long term for the conversion THA group was lower (33.6 ± 13.8) vs. primary THA control (40.9 ± 9.2) (*p* value = 0.008). There was no significant difference in EQ-5D health or pain parameters between groups (*p* value > 0.05). Schnaser et al. [33] showed higher MFA scores in patients who underwent THA conversion for acetabular fracture compared to primary THA [(40 ± 24 vs. 19 ± 12) *p* value = 0.02] (Additional file 1: Table S3).

#### Other outcomes

Operation time (SMD [95% CI] 0.88 [0.61, 1.15]; *p* value < 0.00001; *I*^2^ = 52%) (Additional file 1: Fig. S3A), blood loss (SMD [95% CI] 0.83 [0.56, 1.11]; *p* value < 0.00001; *I*^2^ = 51%) (Additional file 1: Fig. S3B), and bone graft need (OR [95% CI] 27.84 [11.80, 65.65]; *p* value < 0.00001; *I*^2^ = 49%) (Additional file 1: Fig. S3C) were higher in patients undergoing conversion THA. Aali Rezaie et al. [9], Bellabarba et al. [17], and Lee et al. [24] observed in the group of patients undergoing conversion THA, transfusion rates and needs were higher than primary THA (*p* value < 0.001), (*p* value < 0.001), (*p* value < 0.023). No significant difference between the two cohorts was found regarding perioperative transfusion requirements (*p* value = 0.43), Lizaur-Utrilla et al. reported [25]. According to Scott et al. [34], the conversion THA group had a higher likelihood of having an LLD of > 10 mm (long or short) (*p* value = 0.001) (Additional file 1: Table S4).

### Group B: ORIF vs. conservative treatments

#### Complications and revisions

**Fig. 4:**
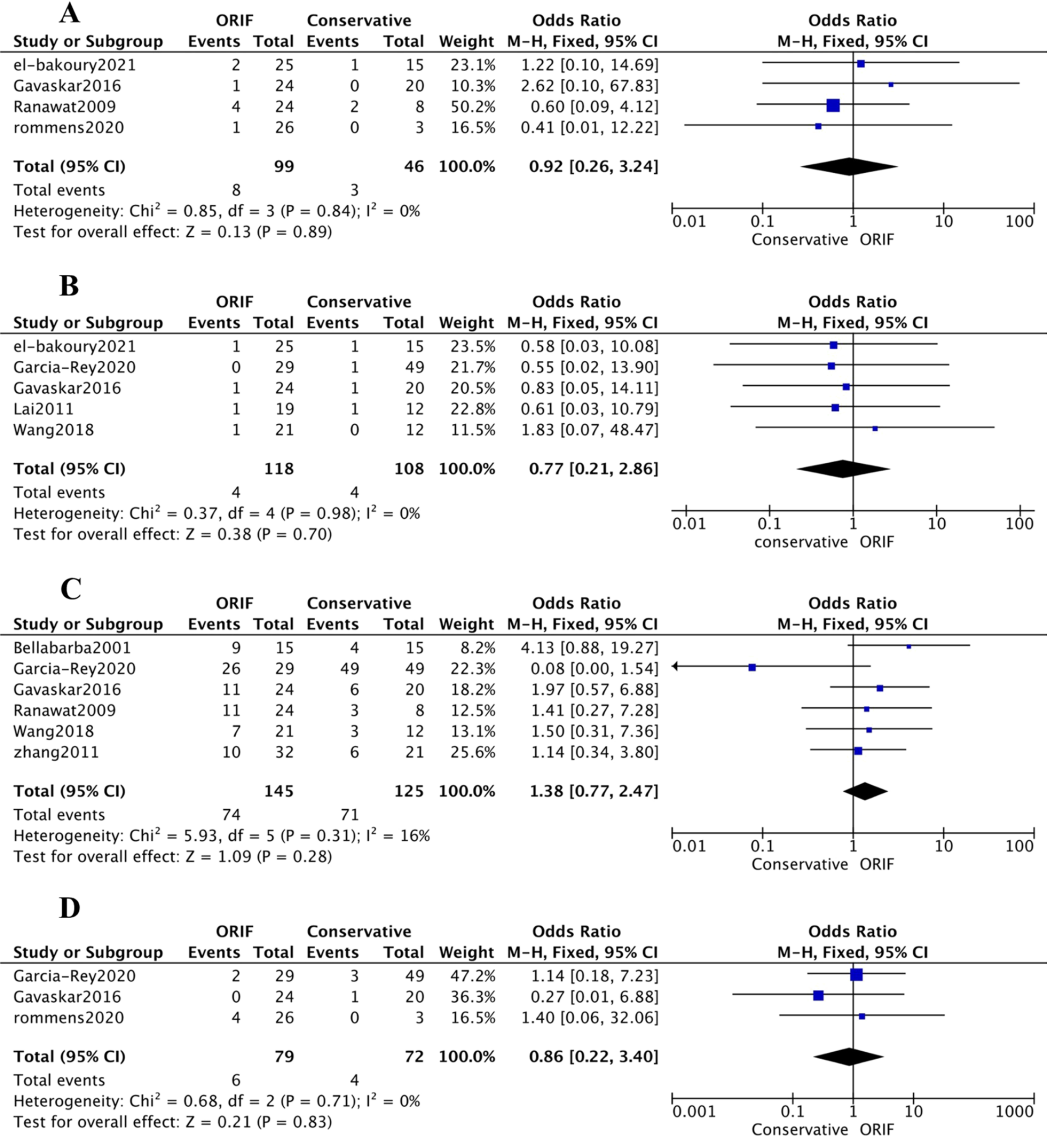
Forest plots demonstrating the rate of infection (**A**), dislocation (**B**), heterotopic ossification (**C**), and implant loosening **D** in those who underwent ORIF versus conservative

The difference between the groups for the following complications was not statistically significant: infection (OR [95% CI] 0.92 [0.26, 3.24] (Fig. 4A); *p* value = 0.89; *I*^2^ = 0%), dislocation (OR [95% CI] 0.77 [0.21, 2.86]; *p* value = 0.7; *I*^2^ = 0%) (Fig. 4B), heterotopic ossification (OR [95% CI] 1.38 [0.77, 2.47]; *p* value = 0.28; *I*^2^ = 16%) (Fig. 4C), and Implant loosening (OR [95% CI] 0.86 [0.22, 3.40]; *p* value = 0.83; *I*^2^ = 0%) (Fig. 4D). Revision (OR [95% CI] 1.64 [0.71, 3.81]; *p* value = 0.25; *I*^2^ = 0%) (Additional file 1: Fig. S4) denoted no association with prior ORIF or conservative treatment for the acetabular fracture. Moreover, García-Rey et al. [21] reported two patients having sciatic palsies in the ORIF group vs. 0 in the conservative treatment group (Additional file 1: Table S2).

#### Functional outcomes

**Fig. 5:**
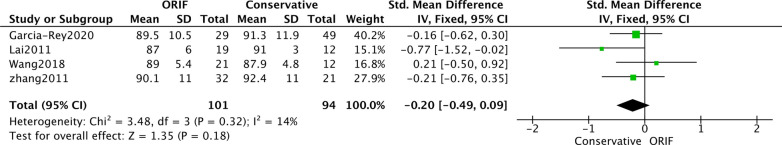
Forest plots demonstrating the port-op HHS in those who underwent ORIF versus conservative

Postoperative HHS (SMD [95% CI] − 0.20 [− 0.49, 0.09]; *p* value = 0.18; *I*^2^ = 14%) (Fig. 5) did not demonstrate a notable difference between patients with previous ORIF or conservative treatments. El-Bakoury et al. [20] found no significant difference in postoperative Median OHS between the ORIF and conservative groups (*p* value = 0.485). García-Rey et al. [21] perceived that the ORIF group had a lower postoperative range of motion at the most recent follow-up (*p* value = 0.05). Postoperative Merle d'Aubigne did not show a difference between the groups in studies García-Rey et al. [21] (Pain, *p* value = 0.265), (Function, *p* value = 0.849) and Gavaskar et al. [11] (Pain, *p* value = 0.2); neither did the reported Oxford hip score by Gavaskar et al. [11] (*p* value = 0.68) and El-Bakoury et al. [20] (*p* value = 0.485). Gavaskar et al. [11] reported a return to work for ORIF and non-ORIF groups to be 16 ± 5 and 23 ± 10 weeks correspondingly (*p* value = 0.004) (Additional file 1: Table S3).

#### Other outcomes

The difference between the groups for operation time (SMD [95% CI] − 0.00 [− 1.12, 1.11]; *p* value = 0.99; *I*^2^ = 87%) (Additional file 1: Fig. S5A) and blood loss (SMD [95% CI] 0.35[− 1.32, 2.02]; *p* value = 0.68; *I*^2^ = 94%) (Additional file 1: Fig. S5B) did not reach statistical significance. Bone graft (OR [95% CI] 0.48 [0.27, 0.86]; *p* value = 0.01; *I*^2^ = 41%) (Additional file 1: Fig. S5C) was computed to be needed more in patients with former acetabular fracture conservative treatment. Bellabarba et al. [17] and Wang et al. [36] found that ORIF patients had more transfusion units than the patients in the closed-treatment group (*p* value = 0.05), (*p* value = 0.00). Gavaskar et al. [11] noticed that compared to the conservative treatment group, patients in the ORIF group needed significantly less blood transfusions (*p* value = 0.03). Lai et al. [23] reported no difference in the amount of transfused blood between 2 groups (*p* value = 0.001).

Gavaskar et al. calculated that the mean limb length discrepancy at follow-up was < 1 cm and it was similar between both groups as in the study by García-Rey et al. (*p* value = 0.063) [21], (*p* value = 0.47) [11] (Additional file 1: Table S4).

### Group C: acute vs. delayed THA

#### Complications and revisions

**Fig. 6:**
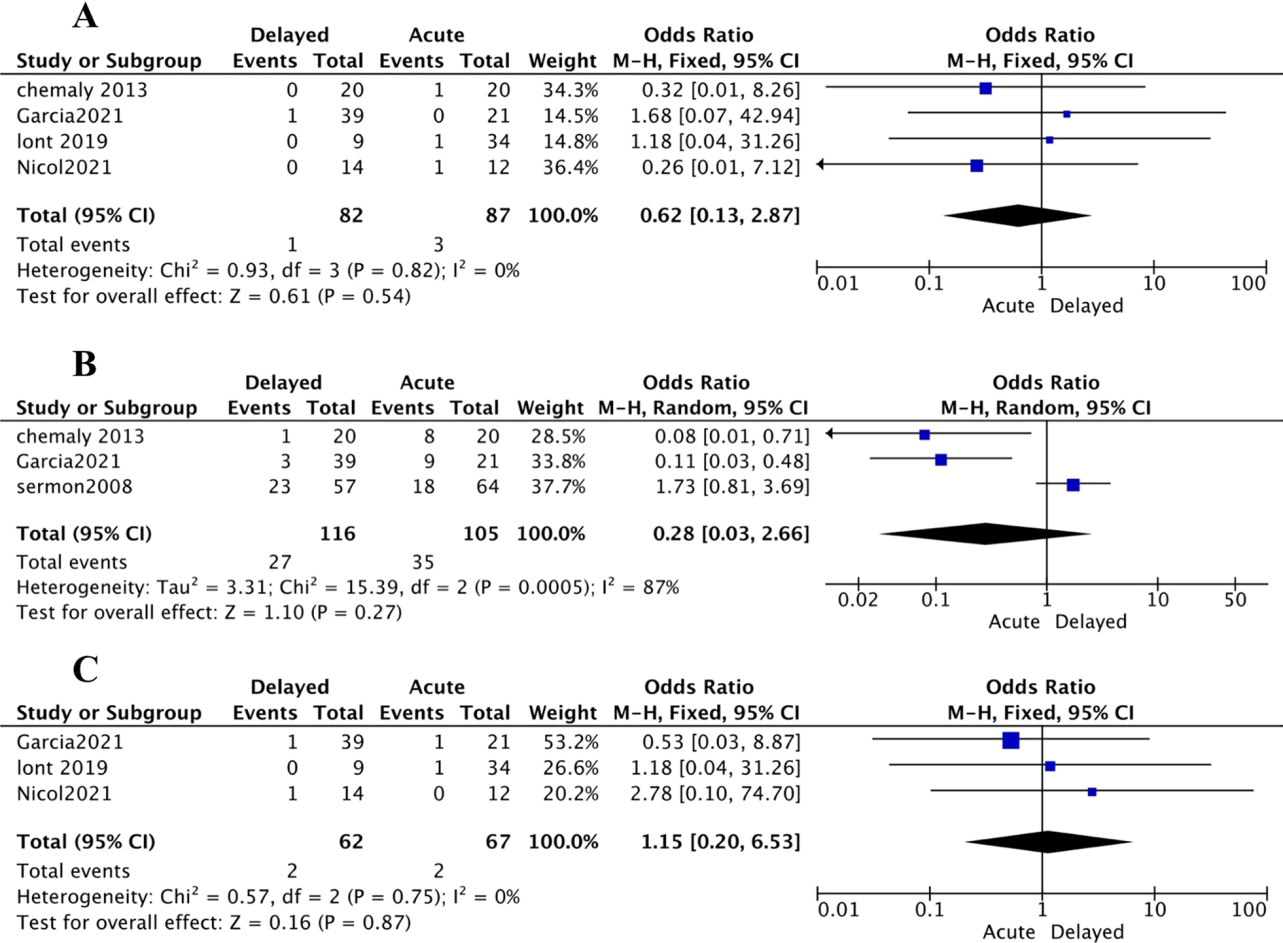
Forest plots demonstrating the rate of dislocation (**A**), heterotopic ossification (**B**), and postoperative fracture **C** in those who underwent acute THA versus delayed THA

The following postoperative complications had no statistically significant relationship with undergoing aTHA or dTHA: dislocation (OR [95% CI] 0.62 [0.13, 2.87]; *p* value = 0.54; *I*^2^ = 0%) (Fig. 6A), heterotopic ossification (OR [95% CI] 0.28 [0.03, 2.66]; *p* value = 0.27; *I*^2^ = 87%) (Fig. 6B), and postoperative fracture (OR [95% CI] 1.15 [0.20, 6.53]; *p* value = 0.87; *I*^2^ = 0%) (Fig. 6C). Although the meta-analysis was not performed (due to lacking enough studies), Chamaly et al. and Garcia et al. observed that aTHA led to a higher incidence of postoperative deep vein thrombosis DVT compared to dTHA (9/41 vs. 1/59 patients) (Additional file 1: Table S2).

#### Functional outcomes

**Fig. 7:**
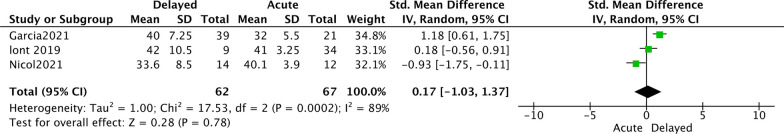
Forest plots demonstrating the OHS in those who underwent acute THA versus delayed THA

Meta-analysis was performed on the mean OHS, yielding no statistically significant association with either aTHA or dTHA group (SMD [95% CI] 0.17 [− 1.03, 1.37]; *p* value = 0.78; *I*^2^ = 89%) (Fig. 7). We could not conduct meta-analysis on the following outcomes due to the paucity of data: As Gracia et al. [22] proclaimed, postoperative HHS was higher in dTHA (*p* value = 0.05); while Merle d'Aubigne was significantly lower in the aTHA group (*p* value = 0.007) [22]. Carroll et al. noted MFA summary, SF-36 Mental component (*p* value = 0.8), SMFA bother (*p* value = 0.3), and SMFA dysfunction (*p* value = 0.7) scores did not show any difference between aTHA and dTHA (*p* value = 0.7); except SF-36 Physical component (*p* value = 0.02) [18] (Additional file 1: Table S3).

#### Other outcomes

Longer operation time (SMD [95% CI] − 1.63 [− 2.73, − 0.53]; *p* value = 0.004; *I*^2^ = 85%) (Additional file 1: Fig. S6A) was noted in patients undergoing aTHA, but no correlation was seen regarding blood loss (SMD [95% CI] − 0.95 [− 2.25, 0.34]; *p* value = 0.15; *I*^2^ = 92%) (Additional file 1: Fig. S6B) between the groups. Gracia et al. computed the mean transfused units to be more in aTHA patients than dTHA (*p* value < 0.001) [22]. Nicol et al. suggested acute THA was not associated with an increase in transfusion requirements (*p* value = 0.3) and that there was an LLD greater than 1 cm after THA in two aTHA patients and five dTHA, yet the association was not statistically significant (*p* value = 0.3) [13] (Additional file 1: Table S4).

### Publication bias

Publication bias was assessed using Egger’s test and Begg’s funnel plot on the outcomes with the largest number of studies: Infection of conversion versus primary THA (*p* value = 0.52), heterotopic ossification of ORIF vs. conservative treatment (*p* value = 0.18), and dislocation of acute vs. delayed THA (*p* value = 0.47). No publication bias was detected in any of the above-mentioned outcomes. (Additional file 1: Fig. S7–9).

## Discussion

**Fig. 8:**
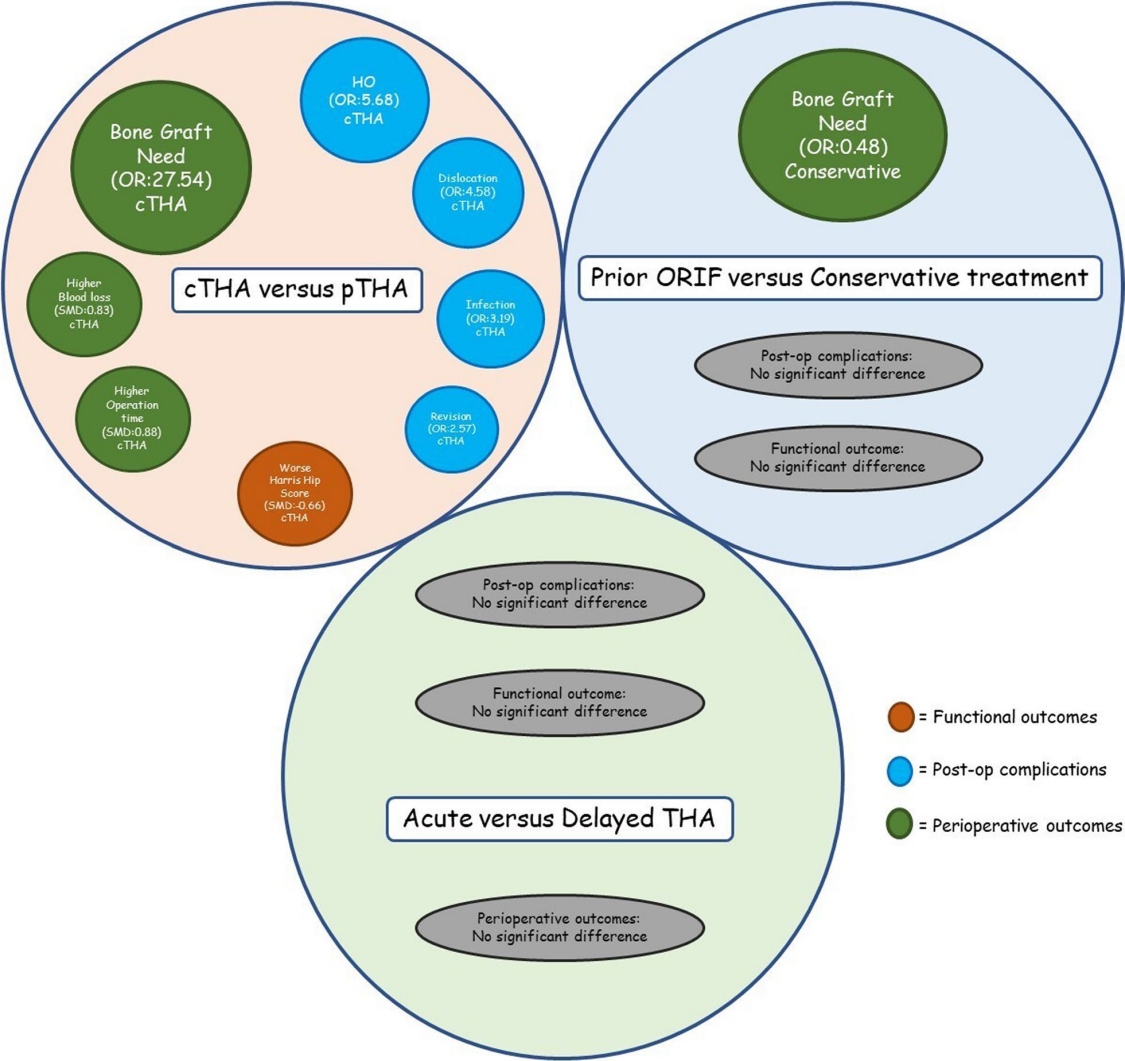
A quick summary of the study results

Providing a strategy to manage failed acetabular fracture fixation has been discussed by many clinicians, but still, serious controversies remain. Around 10–20% rate [38–40] of twenty-year failure in operated acetabular fracture has been reported by previous research highlighting the importance of determining the best salvage procedure. The strengths of conversion THA should be weighed against its potential challenges, such as more complexity of the procedure because of altered hip structure and previous tissue scarring [41]. In this study, we have reviewed current evidence on these issues. Moreover, by dividing the included articles into three groups, we have illustrated differences in functional outcomes and complications between each of these interventions to facilitate the decision-making for the orthopedic surgeons. A summary of our findings is depicted in Fig. 8.

### Primary vs. conversion THA

#### Complications and revision

There was a significantly higher infection rate in conversion THA, which might be attributed to increased operative time, excessive blood loss, and more extensive soft tissue dissection. In addition, the potential role of retained hardware as a source of infection remains controversial [9]. Performing two-stage THA combined with a period of antibiotic therapy between two operations in those with infection marker elevation has been discussed but has yet to be proven to be definitely effective [5, 8]. Furthermore, reducing blood loss via expeditious operation, tranexamic acid use, and hypotensive anesthesia could be a potential way to minimize the infection rate [9].

According to our results, heterotopic ossification was significantly more frequent in the conversion THA group. A previous systematic review [42] illustrated a HO incidence of 30% in conversion THA versus a highly variable incidence rate of 5–90% in primary THA. Although severe HO necessitating intervention remained uncommon in both groups, prophylaxis has been recommended for patients with prior HO following fracture [29, 34].

There was a significantly greater rate of dislocation in conversion THA. Markidis et al. [42] reported a dislocation rate of 4.4% in patients undergoing conversion THA compared to a range of 0.2–7% observed in primary THA. The implant loosening rate did not reach the statistical significance level; however, the sensitivity analysis demonstrated that by excluding Morrison et al.’s study, the implant loosening rate became significantly higher in conversion THA. This might be explained by shorter follow-up durations and higher mean age [25, 28, 29].

Revision is significantly higher in the conversion THA group. Elements like altered anatomy following trauma, a sclerotic bone bed, the loss of acetabular bone stock, and challenges with prior devices may explain this higher failure rate [29]. This accords with the revision rate of 8.66–16.4% in conversion THA compared to 0.72–4.06% in the primary THA group, which was illustrated in a previous meta-analysis [42]. As a considerable finding that requires further investigation, Lizaur et al. [25] demonstrated that there was no relation between non-anatomical hip center placement during reconstruction and revision rate or HHS.

### Functional outcome

Harris Hip Score at the final follow-up was significantly higher in the primary THA group. However, Bellabarba et al. [17] declared that the difference between the two groups did not reach the statistical significance level. That is in agreement with the conclusion of Lee et al.’s paper. There was no significant difference in postop activity level based on the University of California, Los Angeles activity scale (UCLA) [24]. This paper also claimed that by performing medialization technique and preoperative CT-scan in posttraumatic THA, insufficient metal shell coverage and cup positioning difficulty would be addressed, respectively leading to a better functional status postoperatively.

### Other outcomes

Operation time and blood loss were significantly greater in conversion THA compared with primary THA. This could be due to the tissue scar from the previous intervention, challenges of previous hardware removal, and managing structural bone defect and graft placement [17, 28]. Bone graft requirement is notably higher among conversion THAs. This finding was further supported by Markidis et al.’s meta-analysis [42]. It may be beneficial to preoperatively determine which conversion THA cases need additional bone grafts during the procedure. In relation to that, Bellabarba et al. [17] revealed a probable association between radiolucency and the need for bone graft during the operation. Therefore, radiographic indices and signs may be useful.

### THA following ORIF vs. conservative treatment

#### Complications and revision

There was no significant difference between the two groups regarding postoperative infection. It should be considered that this result was not corroborated by the findings of a more recent study conducted by El-bakoury et al. [20]. It might be in consequence of low number of patients in the included studies. It is noteworthy that in addition to aspiration and evaluation of ESR and CRP, performing a staged procedure in terms of culture and removing hardware may be essential to decrease the post-THA infection rate [30].

Considering HO, there was no significant difference between the ORIF and conservative groups. However, in Bellarbarba’s study, HO was about two times more prevalent than the conservative group, which did not reach the significance level of *p* value = 0.05. Surprisingly, this difference did not affect the clinical outcomes, which may be explained by the lower general prevalence of class 3 and 4 HO [17]. In addition, Garcia-Rey et al. suggested that a slightly higher prevalence of HO in the ORIF group may be one factor leading to the lower preoperative functional score and postoperative range of mobility [21].

The implant loosening rate was not significantly different between the two groups. This comparable rate of loosening between these two subgroups of conversion THA could be explained by the fact that the bone defects and altered anatomy of the acetabulum that exist in both groups could similarly lead to suboptimal preparation of bone bed and cup positioning in both conservative and ORIF cases. This further could explain the lower rate of loosening in primary THA compared to conversion THA, as was shown in Scott et al.’s study [34] and accords with the results of our sensitivity analysis comparing this complication between cTHA and pTHA groups.

Dislocation incidence was similar between the two groups. Furthermore, almost all observed cases were managed by closed reduction except for a patient previously treated by ORIF for an acetabular fracture. This patient was managed by reorientating the acetabular component and enlarging the head diameter after the failure of close reduction [11].

### Functional outcome

Functional outcome (postop HHS) was similar in the two groups. Garcia Rey [21] illustrated a significantly better preoperative HHS in the conservative group. It should be considered that the dissimilarity of preoperative status between ORIF and conservative can affect postop outcomes [43].

### Other outcomes

Although it is well known that prior ORIF can lead to a more complex THA procedure and increase intraoperative hip instability [17, 36], it was somewhat surprising that there were no significant differences with respect to operation time and blood loss as main intraoperative outcomes, based on our meta-analysis. A considerable heterogeneity (*I*^2^:87% and 94% for operation time and blood loss, respectively) was observed between studies. One of the included studies, Gavaskar et al. [11], highlighted the effect of fixation of the unreduced posterior wall during THA in the conservative group on blood loss and operation time increment, while others [36] suggested that retained hardware in the ORIF group caused more blood loss and prolonged operation time. This controversy might be the reason of the inconsistency between the study findings.

Our meta-analysis illustrated that bone graft need during the salvage THA is significantly higher in patients who have undergone conservative management as first-line treatment compared to the ORIF group. This can be attributed to the higher bone defect incidence in patients managed with nonoperative treatment prior to THA. This outcome is contrary to that of Zhnag et al. [37], who found that more cases need bone graft during THA in the ORIF group compared to the conservative group. This inconsistency can be explained by the fact that there were more associated fracture patterns in the ORIF group in Zheng et al.’s study, while in others, associated fractures were mainly managed by conservative treatment because of their poor outcomes [11].

### Acute THA vs. delayed THA

#### Complications and revision

There was no significant difference between acute and delayed THA regarding dislocation and post-operation fracture. Moreover, with respect to HO, there was no significant difference between the two groups, and a significant heterogeneity (*I*^2^ = 87%) was observed among studies that were included; nonetheless, Sensitivity analysis showed that by excluding the Sermon et al.’s study, HO incidence would be significantly lower in delayed THA (*p* value = 0.0002 and OR = 0.10), which is consistent with the conclusion of previous reviews [44, 45]. Furthermore, there was no heterogeneity(*I*^2^ = 0%) after excluding the Sermon et al.’s study [35]. This could be related to the fact that the criteria applied for HO incidence in Sermon et al.’s study were incompatible with the other studies (Brooker I–IV in Sermon et al.’s study, Brooker II–IV in others). In addition, utilizing different approaches has been proven to cause a dissimilar expected rate of HO incidence [3]. In Sermon et al.’s investigation, most patients were operated through an anterolateral approach despite the posterior approach in the other two papers. Prophylactic plans, including indomethacin consumption or radiotherapy, might be considered for patients undergoing acute THA [45, 46]. Furthermore, the debridement of necrotic tissue in the gluteal and short external rotator muscles around the posterior portion of the acetabulum may be an alternative option, too [47].

Even without performing a meta-analysis, some complications, such as DVT, occurred more following acute THA. This could be related to the fact that in some previous studies, patients who had undergone acute THA were inclined to be older and had higher ASA scores in comparison with the delayed THA [22]. Performing Doppler ultrasound as a screening test and Taking early VTE prophylaxis (either with anticoagulants or inferior vena cava filter) has been shown to cause considerable reduction of thromboembolic events in pelvic fracture [48–50].

### Functional outcome

Meta-analysis of OHS showed no considerable difference between the two procedures. Nonetheless, there was considerable inter-study heterogeneity (*I*^2^ = 89%). This could be attributed to the different prevalence of uncemented and cemented THAs in each study, as previous investigations have suggested a better outcome of uncemented arthroplasty [22, 35, 51].

Furthermore, preoperative OHS was not reported in any of the included studies. The difference between preoperative and postoperative OHS is a more reliable index to quantify the effectiveness of our intervention, as different preoperative OHS in each study population subgroup can lead to different postoperative OHS [22]. However, this comparison could not be performed, mainly in patients undergoing aTHA.

Garcia et al. [22] explained a better outcome in the delayed THA group by the different fixation methods and more prevalent use of uncemented components in this group. The result of a better outcome in delayed THA is consistent with the slightly better Harris Hip Score in Sermon et al.’s study [35]. On the other hand, Nicol et al. [13] indicated a better OHS in acute THA groups along with more weight-bearing restriction, prolonged hip pain, and more prevalence of leg length discrepancy > 1 cm in the delayed THA group. Their finding is consistent with Carroll et al.’s study [18] result, suggesting a significantly better physical composite of SF-36. According to the fact that aTHA is mostly indicated in older patients [52], age matching between the two groups is of paramount importance.

### Other outcomes

The operation time and blood loss were not significantly different between the two groups; nevertheless, sensitivity analysis illustrated that by excluding Nicol et al.’s study, operation time became significantly shorter in delayed THA. It may be explained by different methods measuring operation time in the delayed THA group, as primary ORIF operation time was added to the delayed THA operation time, which is in contrast to other studies included. The longer operation time of acute THA can be due to the complexity of fracture patterns (most often anatomical reduction is impossible) and the overall higher age of these cases [13]. In addition, acute THA is a combined procedure that necessitates achieving a stabilized construction before performing THA and consequently prolongs intervention [19].

### Limitations

There are several limitations to this review, as follows:

1. In most of the studies, the baseline (pre-treatment) functional score was unavailable, so we could not measure the effect of the intervention over a longitudinal period in a single group.
2. In addition, follow-up durations were not similar between the included studies.
3. There was not a complete inter-study similarity based on population age, which is a potential source of heterogeneity and affects the reliability of some of the investigated outcomes, such as Harris hip score [25] and component loosening [28, 29]. Moreover, acute THA was mostly indicated in old cases, which may be a major source of bias in the comparison of outcomes between acute and delayed THA.
4. There was not a defined standard of THA method to be included in our study, which may affect the precision of our results as there are differences between outcomes of cemented and cementless THAs; as an example, Lizaur et al. [25] claimed that utilizing cementless THA there was no difference between the primary and conversion THA in terms of revision rate, in contrast to our meta-analysis and also Scott et al.’s findings [34].
5. First-line treatment was not the same in all the patients included in the studies. This is a source of selection bias (especially in group A), as nonoperative treatment or percutaneous fixation may be utilized in more complicated patients. In addition, the dissimilarity of preoperative functional status between ORIF and the conservative group may lead to measurement bias.
6. According to the injury to the operation duration, the definitions of acute and delayed THA were not completely similar in the included studies. Moreover, the outcomes of different surgical approaches were not reported in most of the included studies. So, we could not analyze this outcome in our study.

## Conclusions

Comparing cTHA and pTHA, our meta-analysis demonstrated that postoperative infection, HO, dislocation, and revision rate were significantly higher in patients who underwent cTHA. In contrast, implant loosening was not significantly different between these two groups. Moreover, postoperative HHS was significantly higher in the primary THA group. Bone graft need, operation time, and intraoperative blood loss were also higher in conversion THA patients. Comparing ORIF or conservative first-line management, meta-analysis illustrated that the incidence rate of infection, HO, implant loosening, dislocation, and revision were comparable between the groups. Moreover, postoperative HHS, blood loss, and operation time were not significantly different. In contrast, bone graft need was significantly higher in patients with prior conservative treatment. Comparing acute THA and delayed cTHA demonstrated that dislocation, postoperative fracture, and HO incidence rates were not significantly dissimilar between the groups. Postoperative OHS, intraoperative blood loss, and operation time were not significantly distinct between the patients. With regard to other outcomes, there was not sufficient data to perform a meta-analysis. According to this issue, we suggest that performing prospective studies with larger sample size is highly demanded (mainly for the comparison of acute and delayed THA in age-matched groups) to obtain a consensus on differences between different methods of acetabular fracture failure management. With regards to the reviewed recommendations in this paper, to improve the result in groups with worse outcomes, further research could assess the efficacy of these possible solutions to be integrated into current clinical practice.

### Supplementary Information

**Additional file 1 : Fig. S1.** Risk of bias assessment of eligible studies using base on Newcastle Ottawa scale (NOS).** Fig. S2.** Forest plots demonstrating the revision rate in those who underwent cTHA versus pTHA. Fig. S3. Forest plots demonstrating the operation time (**A**), blood loss (**B**), and bone graft need (**C**) in those who underwent cTHA versus pTHA.** Fig. S4.** Forest plots demonstrating the revision rate in those who underwent ORIF versus conservative.** Fig. S5.** Forest plots demonstrating the operation time (**A**), blood loss (**B**), and bone graft need** C** in those who underwent ORIF versus conservative.** Fig. S6.** Forest plots demonstrating the operation time (**A**) and blood loss** B** in those who underwent acute THA versus delayed THA.** Fig. S7.** Funnel plot of the group A studies reporting infection rate.** Fig. S8.** Funnel plot of the group B studies reporting heterotopic ossification.** Fig. S9.** Funnel plot of the group C studies reporting dislocation rate.

## Data Availability

This study was a meta-analysis of previously published studies.
