# Supplementary material for: The outcome of conversion total hip arthroplasty following acetabular fractures: a systematic review and meta-analysis of comparative studies
Source: J Orthop Surg Res. 2024 Jan 20;19:83. doi: 10.1186/s13018-024-04561-x (PMC10799400; doi:10.1186/s13018-024-04561-x)
Supplement: Supplementary file 1 — Additional file 1 : Fig. S1. Risk of bias assessment of eligible studies using base on Newcastle Ottawa scale (NOS). Fig. S2. Forest plots demonstrating the revision rate in those who underwent cTHA versus pTHA. Fig. S3. Forest plots demonstrating the operation time (A), blood loss (B), and bone graft need (C) in those who underwent cTHA versus pTHA. Fig. S4. Forest plots demonstrating the revision rate in those who underwent ORIF versus conservative. Fig. S5. Forest plots demonstrating the operation time (A), blood loss (B), and bone graft need C in those who underwent ORIF versus conservative. Fig. S6. Forest plots demonstrating the operation time (A) and blood loss B in those who underwent acute THA versus delayed THA. Fig. S7. Funnel plot of the group A studies reporting infection rate. Fig. S8. Funnel plot of the group B studies reporting heterotopic ossification. Fig. S9. Funnel plot of the group C studies reporting dislocation rate. [file 13018_2024_4561_MOESM1_ESM.docx]

**The outcome of conversion arthroplasty following acetabular fracture:** **a** **systematic** **review** **and** **meta-analysis of comparative studies**

**Supplemental Material**

**Contents**

**Table S1…………………………………………………………………….Page 3**

**Table S2…………………………………………………………………….Page 4**

**Table S3…………………………………………………………………….Page 6**

**Table S4…………………………………………………………………….Page 8**

**Figure S1…………………………………………………...........................Page 10**

**Figure S2…………………………………………………………………...Page 11**

**Figure S3…………………………………………………...........................Page 12**

**Figure S4…………………………………………………...........................Page 13**

**Figure S5…………………………………………………...........................Page 14**

**Figure S6…………………………………………………...........................Page 15**

**Figure S7…………………………………………………...........................Page 16**

**Figure S8…………………………………………………...........................Page 17**

**Figure S9…………………………………………………...........................Page 18**

| **Table S1.** Complete search strategy | | |
| --- | --- | --- |
| Database | Search string | Number of results |
| Pubmed | (("Fracture Fixation"[Mesh] OR "fracture reduction"[title/abstract] OR "fracture fixation"[title/abstract] OR "Orthopedic Fixation Devices"[Mesh] OR ORIF[title/abstract] OR "Open Reduction and Internal Fixation"[title/abstract]) AND ("Acetabulum"[Mesh] OR Acetabul*[title/abstract])) AND ("Arthroplasty, Replacement, Hip"[Mesh] OR "Total Hip Replacement"[Title/Abstract] OR THR[title/abstract] OR "Total hip arthroplasty"[title/abstract] OR THA[title/abstract] OR "Hip Prosthesis"[Title/Abstract] OR "Hip Prosthesis Implantation"[Title/Abstract] OR "arthroplasty"[Title/Abstract] OR "hemiarthroplasty"[Title/Abstract]) | 809 |
| Scopus | (TITLE-ABS-KEY(''fracture reduction'') OR TITLE-ABS-KEY(''fracture fixation'') OR TITLE-ABS-KEY(''Orthopedic Fixation Devices'') OR TITLE-ABS-KEY(''Open Reduction and Internal Fixation'') OR TITLE-ABS-KEY(''ORIF'')) AND (TITLE-ABS-KEY(''Acetabulum'') OR TITLE-ABS-KEY(Acetabul*)) AND (TITLE-ABS-KEY(''Arthroplasty, Replacement, Hip'') OR TITLE-ABS-KEY(''Arthroplasty'') OR TITLE-ABS-KEY(''Hemiarthroplasty'') OR TITLE-ABS-KEY(''Total Hip Replacement'') OR TITLE-ABS-KEY(''THR'') OR TITLE-ABS-KEY(''Total hip arthroplasty'') OR TITLE-ABS-KEY(''THA'') OR TITLE-ABS-KEY(''Hip Prosthesis'') OR TITLE-ABS-KEY(''Hip Prosthesis Implantation'')) | 1953 |
| Web Of Science (WOS) | TS=("Fracture Fixation" OR "fracture reduction" OR "Orthopedic Fixation Devices" OR "ORIF" OR "Open Reduction and Internal Fixation") AND TS=("Acetabulum" OR "Acetabul*") AND TS=("Arthroplasty, Replacement, Hip" OR "Total Hip Replacement" OR "THR" OR "Total hip arthroplasty" OR "THA" OR "Hip Prosthesis" OR "Hip Prosthesis Implantation" OR "arthroplasty" OR "hemiarthroplasty") | 244 |
| Cochrane | ((''fracture reduction'' OR ''fracture fixation'' OR ''Orthopedic Fixation Devices'' OR ''Open Reduction and Internal Fixation'' OR ''ORIF'') AND (''Acetabulum'' OR Acetabul*) AND (''Arthroplasty, Replacement, Hip'' OR ''Total Hip Replacement'' OR ''THR'' OR ''Total hip arthroplasty'' OR ''Arthroplasty'' OR ''Hemiarthroplasty'' OR ''THA'' OR ''Hip Prosthesis'' OR ''Hip Prosthesis Implantation'')):ti,ab,kw | 36 |

| **Table S2.** Postoperative complications following THA after failed acetabular fracture fixation | | | | | | | | | |
| --- | --- | --- | --- | --- | --- | --- | --- | --- | --- |
| **Study** | **Groups** | **Infection** | **Aseptic loosening** | **Dislocation** | **Periprosthetic fracture** | **Nerve injury** | **DVT** | **Revision** | **Heterotopic Ossification** |
| Aalirezaei et al. 2020 | cTHA | 5 | NA | NA | NA | 6 sciatic | NA | NA | NA |
|  | pTHA | 1 | NA | NA | NA | 0 sciatic | NA | NA | NA |
| Bellabarba et al. 2001 | cTHA | NA | 1 | NA | NA | NA | NA | 1 | 13 |
|  | pTHA | NA | 0 | NA | NA | NA | NA | 0 | 10 |
| Lizaur-utrilla et al. 2012 | cTHA | 1 | 1 | 1 | NA | NA | NA | 4 | 2 |
|  | pTHA | 0 | 2 | 0 | NA | NA | NA | 3 | 0 |
| Schnaser et al. 2014 | cTHA | NA | NA | 2 | NA | NA | 2 | 1 | NA |
|  | pTHA | 1 | NA | 0 | NA | NA | 1 | 0 | NA |
| Scott et al. 2017 | cTHA | 2 | 4 | 2 | 1 | NA | NA | 6 | 18 |
|  | pTHA | 4 | 1 | 1 | 2 | NA | NA | 0 | 9 |
| McGowan et al. 2019 | cTHA | 2 | 1 | NA | 1 | NA | NA | 2 | 1 |
|  | pTHA | 0 | 0 | NA | 0 | NA | NA | 0 | 0 |
| Manirajan et al. 2022 | cTHA | 21 | NA | NA | NA | NA | 12 | 13 | NA |
|  | pTHA | 465 | NA | NA | NA | NA | 408 | 320 | NA |
| Morison et al. 2015 | cTHA | 5 | 14 | 8 | 1 | NA | NA | 24 | 32 |
|  | pTHA | 0 | 11 | 2 | 0 | NA | NA | 12 | 12 |
| Lee et al. 2019 | cTHA | NA | NA | 1 | NA | NA | NA | 0 | NA |
|  | pTHA | NA | NA | 0 | NA | NA | NA | 0 | NA |
| Garcia-Rey et al. 2020 | ORIF | 0 | 2 | 0 | NA | 2 | NA | 2 | 24 |
|  | Conservative | 0 | 3 | 1 | NA | 0 | NA | 3 | 33 |
| Gavaskar et al. 2016 | ORIF | 1 | 0 | 1 | NA | NA | 0 | 1 | 11 |
|  | Conservative | 0 | 1 | 1 | NA | NA | 2 | 2 | 6 |
| Lai et al. 2011 | ORIF | 0 | 0 | 1 | NA | 1 sciatic | NA | 0 | NA |
|  | Conservative | 0 | 0 | 1 | NA | 0 sciatic | NA | 0 | NA |
| Ranawat et al. 2009 | ORIF | 4 | NA | NA | NA | NA | NA | NA | 11 |
|  | Conservative | 2 | NA | NA | NA | NA | NA | NA | 3 |
| Wang et al. 2018 | ORIF | NA | 0 | 1 | NA | NA | NA | 2 | 7 |
|  | Conservative | NA | 0 | 0 | NA | NA | NA | 1 | 3 |
| Bellabarba et al. 2001 | ORIF | NA | NA | NA | NA | NA | NA | NA | 9 |
|  | Conservative | NA | NA | NA | NA | NA | NA | NA | 4 |
| Zhang et al. 2011 | ORIF | NA | NA | NA | NA | NA | NA | NA | 10 |
|  | Conservative | NA | NA | NA | NA | NA | NA | NA | 6 |
| Rommens et al. 2020 | ORIF | 1 | 4 | NA | NA | NA | NA | 11 | NA |
|  | Conservative | 0 | 0 | NA | NA | NA | NA | 0 | NA |
| Morison et al. 2015 | ORIF | NA | NA | NA | NA | NA | NA | 21 | NA |
|  | Conservative | NA | NA | NA | NA | NA | NA | 3 | NA |
| El-bakoury et al. 2021 | ORIF | 2 | NA | 1 | NA | 1 | NA | 2 | NA |
|  | Conservative | 1 | NA | 1 | NA | 1 | NA | 0 | NA |
| Nicol et al. 2021 | Acute | 1 | NA | 1 | 0 | NA | NA | NA | 12 |
|  | Delayed | 0 | NA | 0 | 1 | NA | NA | NA | 14 |
| Garcia et al. 2021 | Acute | 1 | 2 | 0 | 1 | NA | 6 | NA | 9 |
|  | Delayed | 4 | 2 | 1 | 1 | NA | 0 | NA | 3 |
| Sermon et al. 2008 | Acute | NA | NA | NA | NA | NA | NA | 4 | 18 |
|  | Delayed | NA | NA | NA | NA | NA | NA | 12 | 23 |
| Chemaly et al. 2012 | Acute | NA | NA | 1 | NA | NA | 3 | NA | 12 |
|  | Delayed | NA | NA | 0 | NA | NA | 1 | NA | 13 |
| Lont et al. 2019 | Acute | 0 | NA | 1 | 1 | NA | NA | NA | NA |
|  | Delayed | 2 | NA | 0 | 0 | NA | NA | NA | NA |
| **Abbreviations:** *THA, Total Hip Arthroplasty; cTHA, Conversion THA; pTHA, Primary THA; DVT, Deep Vein Thrombosis; NA, Not Available* | | | | | | | | | |

| Table S3. Functional status and quality of life after THA following failed acetabular fractures | | | | | | | | | |
| --- | --- | --- | --- | --- | --- | --- | --- | --- | --- |
| Study | **Groups** | **SF-36 PCS** | **SF-36 MCS** | **UCLA** | **EQ5D5L** | **OHS** | **HHS** | **Merle D’Aubigné** | **MFA score** |
| Bellabarba et al. 2001 | cTHA | NA | NA | NA | NA | NA | 88±13.3 | NA | NA |
|  | pTHA | NA | NA | NA | NA | NA | 90 | NA | NA |
| Lizaur-utrilla et al. 2012 | cTHA | NA | NA | NA | NA | NA | 77±16.5 | NA | NA |
|  | pTHA | NA | NA | NA | NA | NA | 88±11.3 | NA | NA |
| Schnaser et al. 2014 | cTHA | NA | NA | NA | NA | NA | 70±25 | NA | 40±24 |
|  | pTHA | NA | NA | NA | NA | NA | 90±6 | NA | 19±12 |
| Scott et al. 2017 | cTHA | NA | NA | NA | Pain:  74.2±20.7  Health:  75.6±19.3 | 33.6±13.8 | NA | NA | NA |
|  | pTHA | NA | NA | NA | Pain:  75.5±31.7  Health:  79.4±20.3 | 40.9±9.2 | NA | NA | NA |
| Lee et al. 2019 | cTHA | NA | NA | 4.9±1.9 | NA | NA | NA | NA | NA |
|  | pTHA | NA | NA | 5.2±2.0 | NA | NA | NA | NA | NA |
| Garcia-Rey et al. 2020 | ORIF | NA | NA | NA | NA | NA | 89.5±10.5 | Pain: 5.8±0.9  Function: 5.4±1.2 | NA |
|  | Conservative | NA | NA | NA | NA | NA | 91.3±11.9 | Pain: 5.8±0.9  Function: 5.6±0.9 | NA |
| Gavaskar et al. 2016 | ORIF | NA | NA | NA | NA | 41.9 ± 3.1 | NA | Pain: 15.1±1.7 | NA |
|  | Conservative | NA | NA | NA | NA | 41.5 ± 3.6 | NA | Pain: 14.5±1.5 | NA |
| Lai et al. 2011 | ORIF | NA | NA | NA | NA | NA | 87±6 | NA | NA |
|  | Conservative | NA | NA | NA | NA | NA | 91±3 | NA | NA |
| Ranawat et al. 2009 | ORIF | NA | NA | NA | NA | NA | 84 | NA | NA |
|  | Conservative | NA | NA | NA | NA | NA | 74 | NA | NA |
| Wang et al. 2018 | ORIF | NA | NA | NA | NA | NA | 89±5.4 | NA | NA |
|  | Conservative | NA | NA | NA | NA | NA | 87.9±4.8 | NA | NA |
| Zhang et al. 2011 | ORIF | NA | NA | NA | NA | NA | 90.1±11 | NA | NA |
|  | Conservative | NA | NA | NA | NA | NA | 92.4±11 | NA | NA |
| El-bakoury et al. 2021 | ORIF | NA | NA | NA | NA | 40 (39-43.2) | NA | NA | NA |
|  | Conservative | NA | NA | NA | NA | 41 (39.25-44) | NA | NA | NA |
| Nicol et al. 2021 | Acute | NA | NA | NA | NA | 40.1±3.9 | NA | NA | NA |
|  | Delayed | NA | NA | NA | NA | 33.6±8.5 | NA | NA | NA |
| Garcia et al. 2021 | Acute | NA | NA | NA | NA | 32±5.5 | 73±11.8 | 12 (8-17) | NA |
|  | Delayed | NA | NA | NA | NA | 40±7.25 | 85±8.8 | 16 (10-17) | NA |
| Carroll et al. 2010 | Acute | 57.9 ± 7.6 | 57.9 ± 7.6 | NA | NA | NA | NA | NA | 22 |
|  | Delayed | 45.9 ± 9.7 | 51.61 ±0.7 | NA | NA | NA | NA | NA | 28.3± 24.8 |
| Lont et al. 2019 | Acute | NA | NA | NA | NA | 41±3.25 | NA | NA | NA |
|  | Delayed | NA | NA | NA | NA | 42±10.5 | NA | NA | NA |
| Abbreviations: *THA, Total Hip Arthroplasty; cTHA, Conversion THA; pTHA, Primary THA; PCS, Physical Component Score; SF, Short Form; MCS, Mental Component Score; UCLA, University of California, Los Angeles; EQ5D5L, European Quality of Life 5 Dimension 5 Level; OHS, Oxford Hip Score; HHS, Harris Hip Score; MFA, Musculoskeletal Functional Assessment; NA, Not Available* | | | | | | | | | |

| **Table S4.** Other perioperative and postoperative outcomes of conversion THA | | | | | | | | |
| --- | --- | --- | --- | --- | --- | --- | --- | --- |
| **Study** | **Groups** | **Operation time, min** | **Blood loss, ml** | **Transfusion need** | **Bone graft need, n** | **LLD, mm** | **Readmission, n** | **LOS, day** |
| Aalirezaei et al. 2020 | cTHA | 153±82 | 483±539 | NA | 12 | NA | NA | NA |
|  | pTHA | 113±40 | 216±141 | NA | 0 | NA | NA | NA |
| Bellabarba et al. 2001 | cTHA | 179±52.5 | 898±662.5 | 2.2 (0-5) unit | 9 | NA | NA | NA |
|  | pTHA | 122±63.5 | 413±418.8 | 1.3 (0-4) unit | 8 | NA | NA | NA |
| Lizaur-utrilla et al. 2012 | cTHA | 81±7.8 | NA | 4 n | 15 | NA | NA | NA |
|  | pTHA | 72.7±6.5 | NA | 7 n | 0 | NA | NA | NA |
| Schnaser et al. 2014 | cTHA | NA | 668±547 | NA | NA | NA | NA | NA |
|  | pTHA | NA | 270±230 | NA | NA | NA | NA | NA |
| Scott et al. 2017 | cTHA | NA | NA | NA | 13 | -3.4 (-37 to 18) | NA | NA |
|  | pTHA | NA | NA | NA | 0 | -0.8 | NA | NA |
| McGowan et al. 2019 | cTHA | 217.4±91.4 | 875.8±616 | NA | 10 | NA | NA | 3.7 (1-8) |
|  | pTHA | 113.7±20.9 | 365±218.3 | NA | 0 | NA | NA | 3.6 (2-7) |
| Manirajan et al. 2022 | cTHA | NA | NA | NA | NA | NA | 42 | NA |
|  | pTHA | NA | NA | NA | NA | NA | 2451 | NA |
| Lee et al. 2019 | cTHA | 145.3±41.7 | 795.6±587.8 | 752.3±684.7 ml | NA | NA | NA | 13.4±8.2 |
|  | pTHA | 123.7±42.2 | 623.7±325.2 | 486.2±553.8 ml | NA | NA | NA | 12.8±5.8 |
| Garcia-Rey et al. 2020 | ORIF | NA | NA | NA | 4 | < 10 mm: 19n  10–20 mm: 8n  20–30 mm: 2n  > 30 mm: 0n | NA | NA |
|  | Conservative | NA | NA | NA | 12 | < 10 mm: 43n  10–20 mm: 4n  20–30 mm: 2n  > 30 mm: 1n | NA | NA |
| Gavaskar et al. 2016 | ORIF | 86±24 | 448±105 | 6 n | 15 | 5.3±4.2 | NA | NA |
|  | Conservative | 115±38 | 652±212 | 13 n | 11 | 6.2±3.9 | NA | NA |
| Lai et al. 2011 | ORIF | 138±29 | 726±288 | 1130±437 ml | 6 | NA | NA | NA |
|  | Conservative | 98±16 | 525±101 | 1016±422 ml | 8 | NA | NA | NA |
| Ranawat et al. 2009 | ORIF | NA | 721 | NA | NA | NA | NA | NA |
|  | Conservative | NA | 711 | NA | NA | NA | NA | NA |
| Wang et al. 2018 | ORIF | 189±57 | 1289±429 | 6.6 unit | NA | NA | NA | NA |
|  | Conservative | 143±32 | 750±145 | 3.5 unit | NA | NA | NA | NA |
| Bellabarba et al. 2001 | ORIF | 202 | 1150 | 2.7 unit | 2 | NA | NA | NA |
|  | Conservative | 157 | 647 | 1.7 unit | 7 | NA | NA | NA |
| Zhang et al. 2011 | ORIF | NA | NA | NA | 17 | NA | NA | NA |
|  | Conservative | NA | NA | NA | 9 | NA | NA | NA |
| Rommens et al. 2020 | ORIF | NA | NA | NA | NA | NA | NA | 16 |
|  | Conservative | NA | NA | NA | NA | NA | NA | 8 |
| Nicol et al. 2021 | Acute | 238±85 | 980±512 | 1.3 (0-4) unit | NA | > 10 mm: 2n | NA | 15±12 |
|  | Delayed | 416±142 | 1127±595 | 1.8 (0-3) unit | NA | > 10 mm: 5n | NA | 23±10 |
| Garcia et al. 2021 | Acute | 137±22.75 | 1145±300 | 4 (2-7) unit | 4 | NA | NA | NA |
|  | Delayed | 92±19.8 | 560±175 | 1.9 (0-5) unit | 14 | NA | NA | NA |
| Chemaly et al. 2012 | Acute | 171±52.5 | 992±425 | NA | NA | NA | NA | NA |
|  | Delayed | 76±52.3 | 416±325 | NA | NA | NA | NA | NA |
| Lont et al. 2019 | Acute | 169±53.3 | 1100±575 | NA | NA | NA | NA | NA |
|  | Delayed | 143±42.3 | 1100±325 | NA | NA | NA | NA | NA |
| **Abbreviations:** *THA, Total Hip Arthroplasty; cTHA, Conversion THA; pTHA, Primary THA; LLD, Leg Length Discrepancy; LOS, Length Of Stay; NA, Not Available;, n, number* | | | | | | | | |


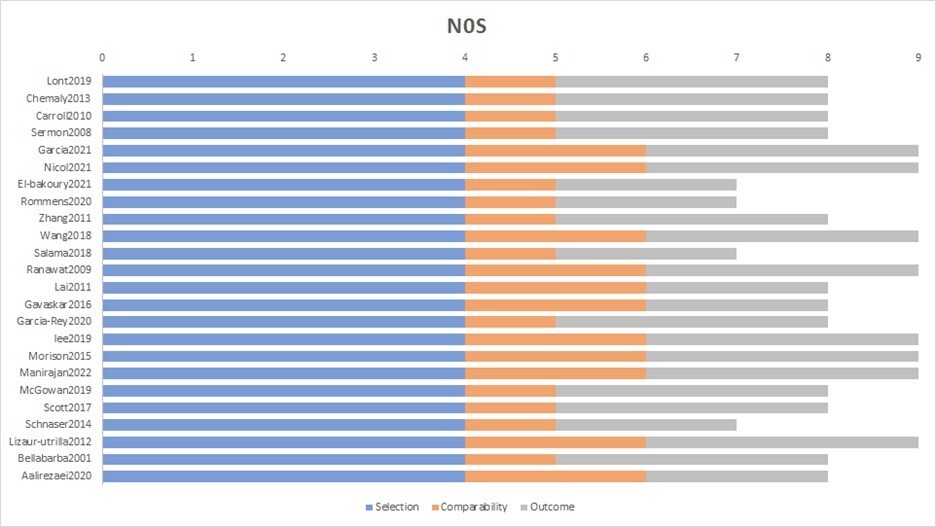


**Figure S1.** Risk of bias assessment of eligible studies using base on Newcastle Ottawa scale (NOS)


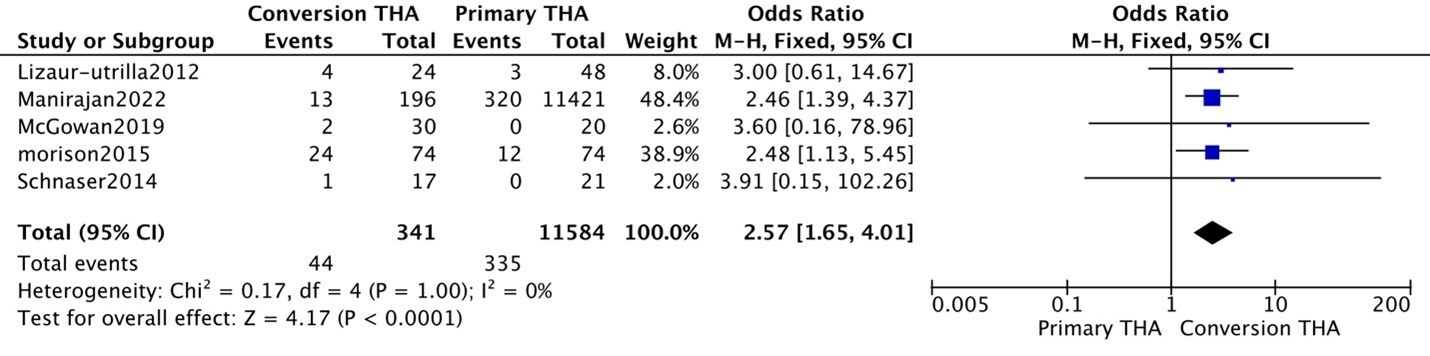


**Figure S2.** Forest plots demonstrating the revision rate in those who underwent cTHA versus pTHA


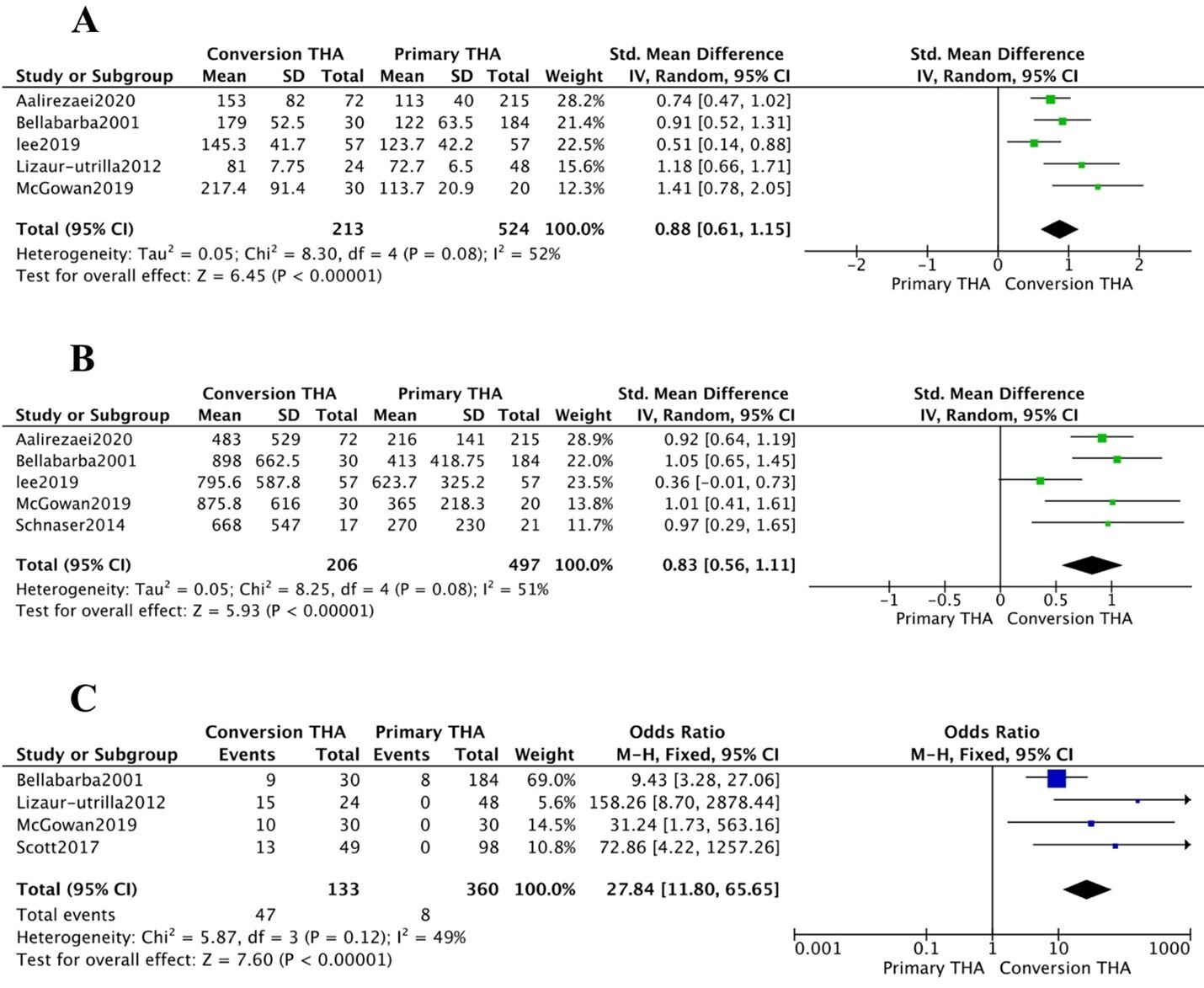


**Figure S3.** Forest plots demonstrating the operation time (A), blood loss (B), and bone graft need (C) in those who underwent cTHA versus pTHA


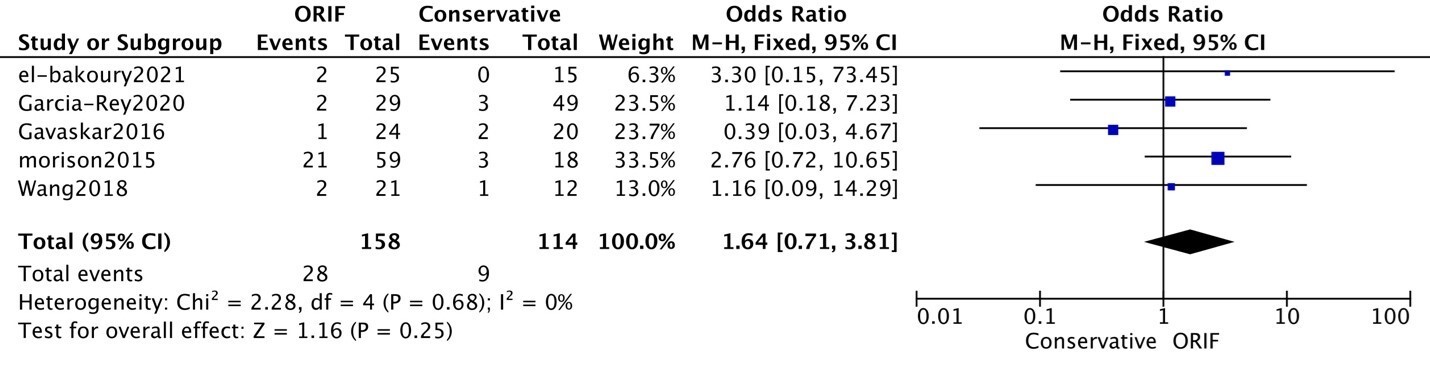


**Figure S4.** Forest plots demonstrating the revision rate in those who underwent ORIF versus conservative


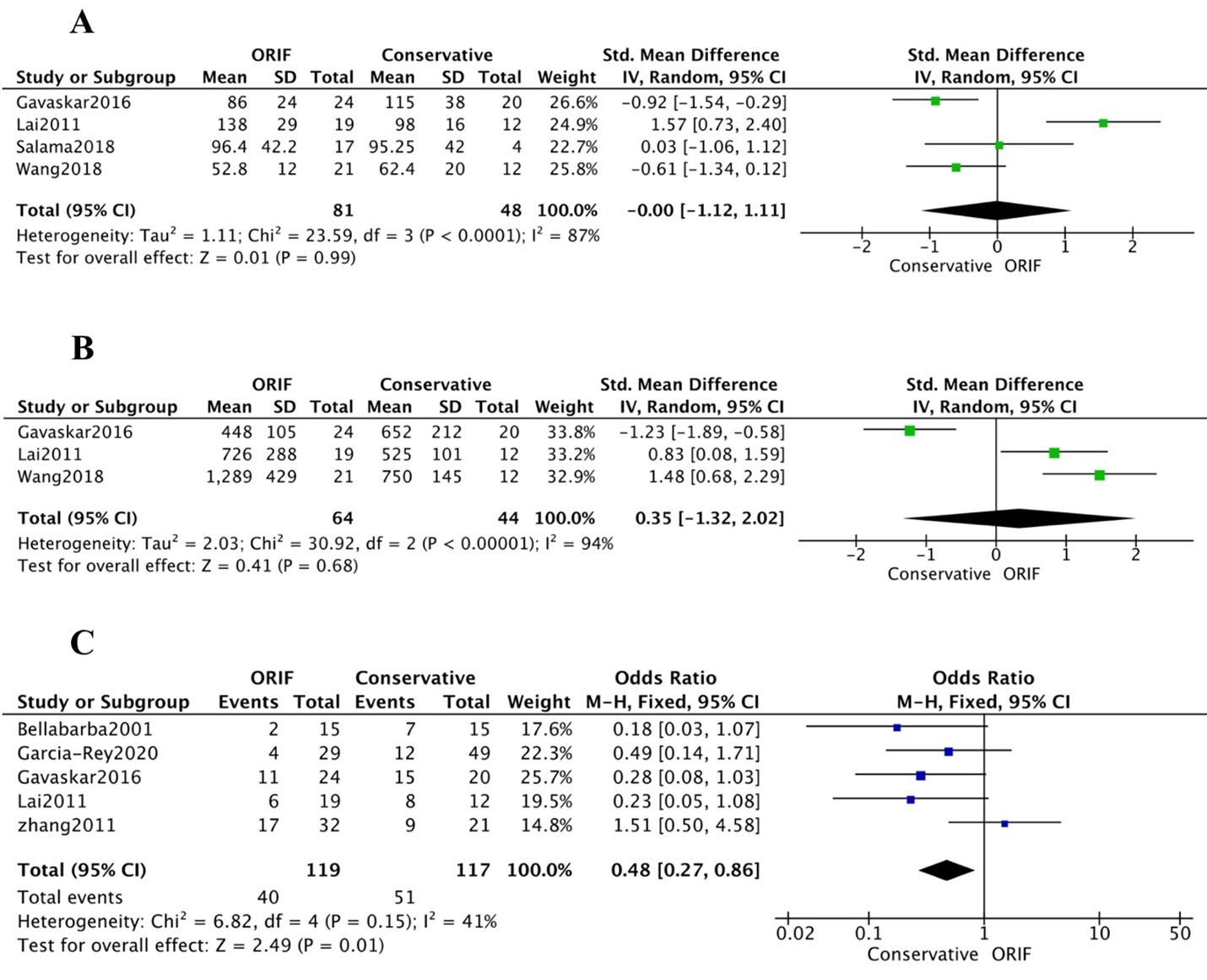


**Figure S5.** Forest plots demonstrating the operation time (A), blood loss (B), and bone graft need (C) in those who underwent ORIF versus conservative


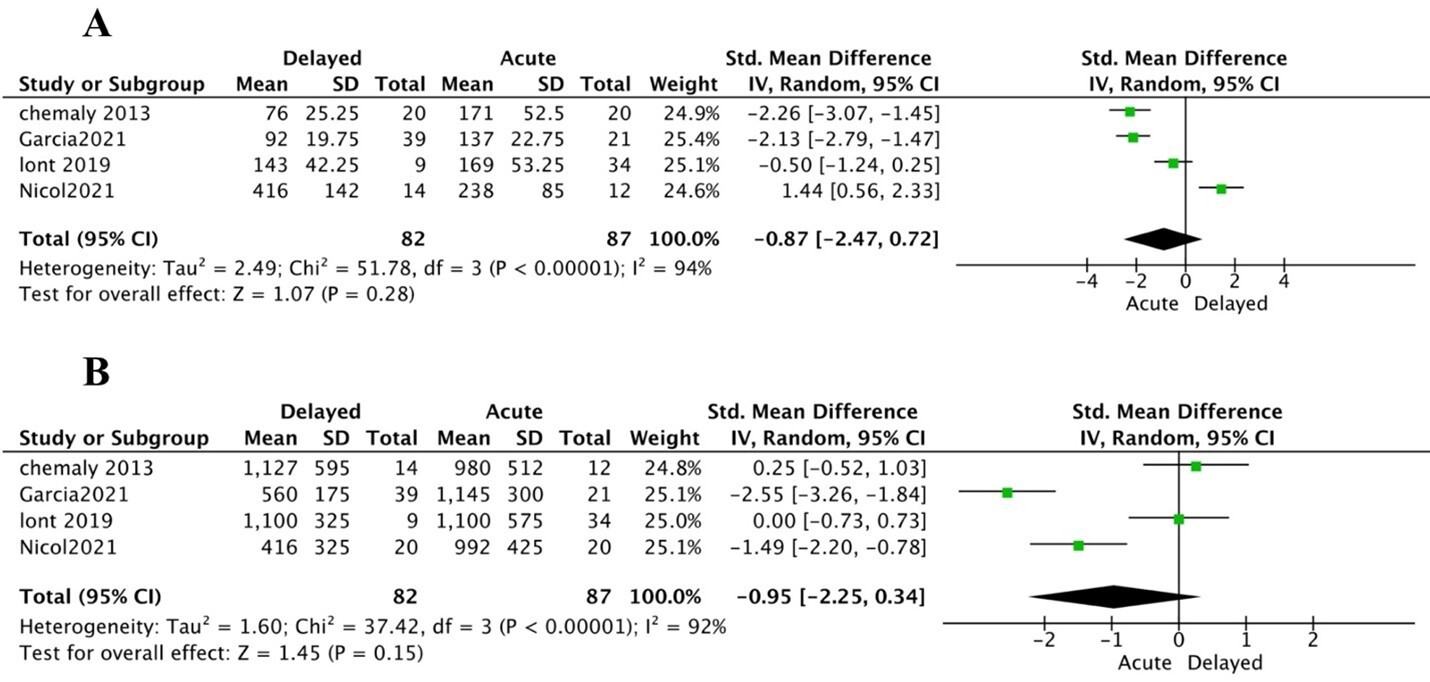


**Figure S6.** Forest plots demonstrating the operation time (A) and blood loss (B) in those who underwent acute THA versus delayed THA


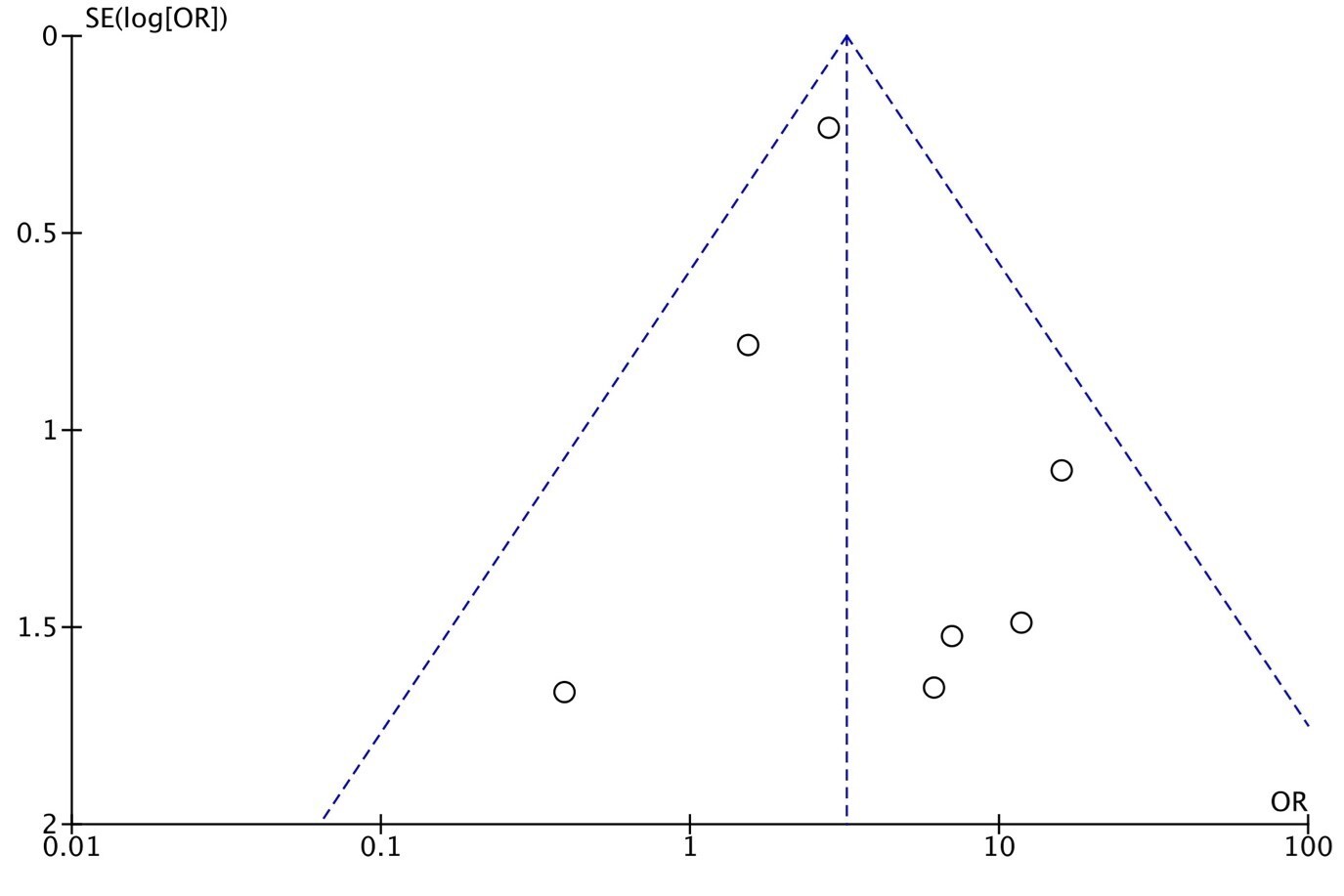


**Figure S7.** Funnel plot of the group A studies reporting infection rate


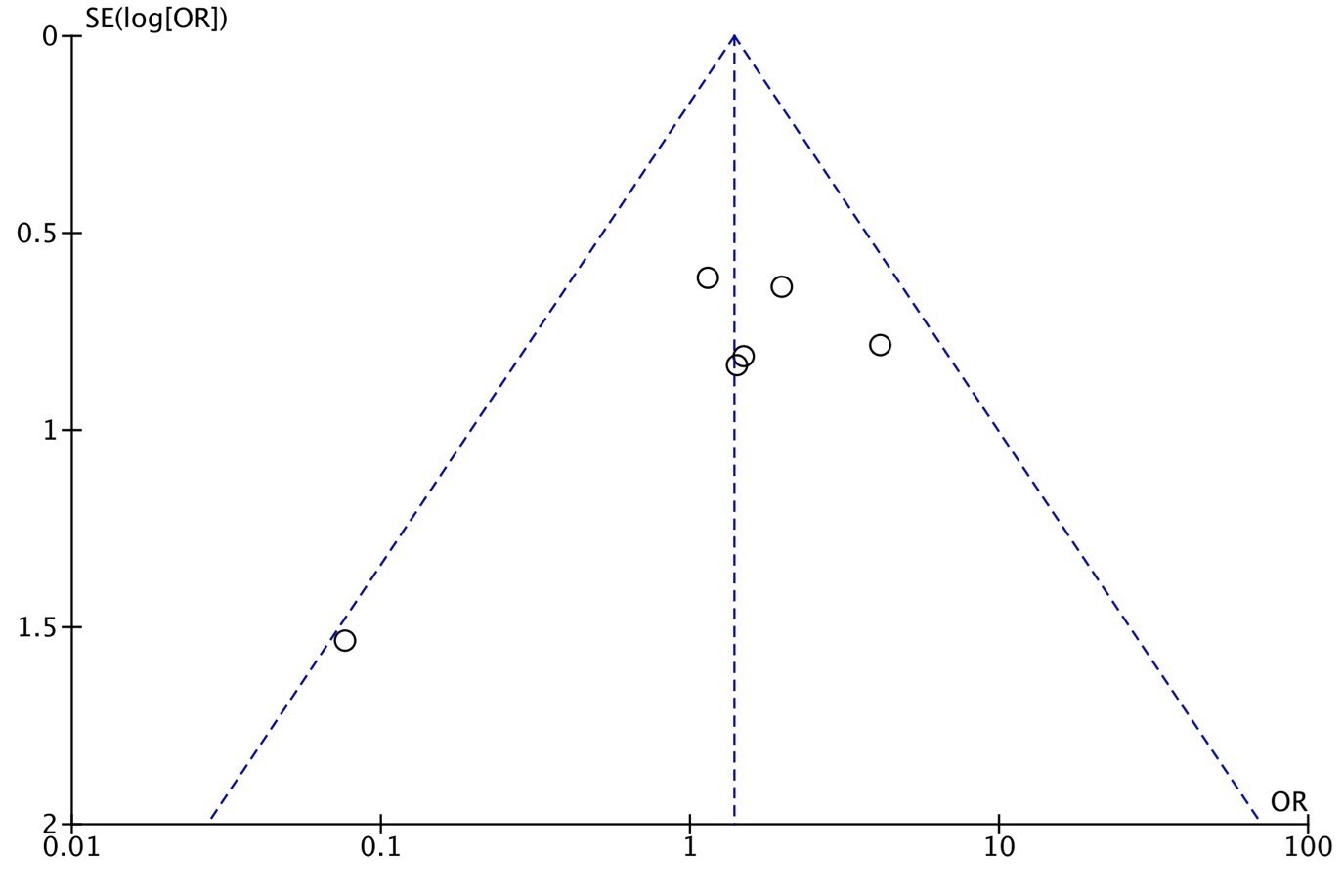


**Figure S8.** Funnel plot of the group B studies reporting heterotopic ossification


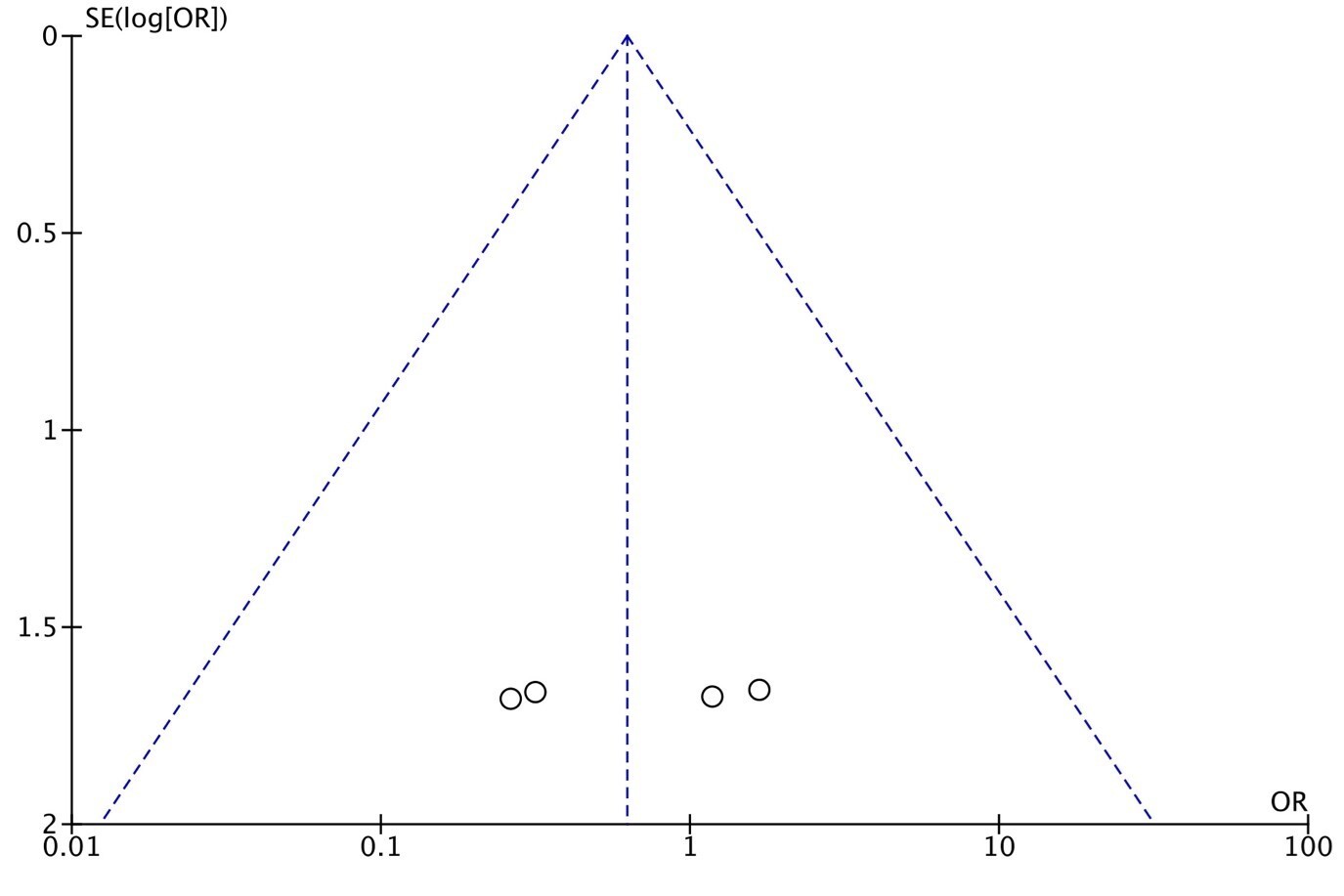


**Figure S9.** Funnel plot of the group C studies reporting dislocation rate
